# Learning to synthesize: robust phase retrieval at low photon counts

**DOI:** 10.1038/s41377-020-0267-2

**Published:** 2020-03-09

**Authors:** Mo Deng, Shuai Li, Alexandre Goy, Iksung Kang, George Barbastathis

**Affiliations:** 1 Department of Electrical Engineering and Computer Science, Massachusetts Institute of Technology, Cambridge, MA 02139 USA; 2 Sensebrain Technology Limited LLC, 2550 N 1st Street, Suite 300, San Jose, CA 95131 USA; 3 Omnisens SA, Riond Bosson 3, 1110 Morges, VD Switzerland; 4 Department of Mechanical Engineering, Massachusetts Institute of Technology, Cambridge, MA 02139 USA; 5 Singapore-MIT Alliance for Research and Technology (SMART) Centre, Singapore, 117543 Singapore

**Keywords:** Applied optics, Imaging and sensing

## Abstract

The quality of inverse problem solutions obtained through deep learning is limited by the nature of the priors learned from examples presented during the training phase. Particularly in the case of quantitative phase retrieval, spatial frequencies that are underrepresented in the training database, most often at the high band, tend to be suppressed in the reconstruction. Ad hoc solutions have been proposed, such as pre-amplifying the high spatial frequencies in the examples; however, while that strategy improves the resolution, it also leads to high-frequency artefacts, as well as low-frequency distortions in the reconstructions. Here, we present a new approach that learns separately how to handle the two frequency bands, low and high, and learns how to synthesize these two bands into full-band reconstructions. We show that this “learning to synthesize” (LS) method yields phase reconstructions of high spatial resolution and without artefacts and that it is resilient to high-noise conditions, e.g*.*, in the case of very low photon flux. In addition to the problem of quantitative phase retrieval, the LS method is applicable, in principle, to any inverse problem where the forward operator treats different frequency bands unevenly, i.e*.*, is ill-posed.

## Image reconstruction: teaching algorithms to look high and low

A computational approach that avoids artifacts when extracting data about the phase of light from noisy intensity signals can improve imaging of transparent objects including cells. Mo Deng from the Massachusetts Institute of Technology, United States, and colleagues have developed a procedure that separates raw intensity signals into high-frequency and low-frequency spectral channels. Then deep neural network algorithms, trained to operate in these two frequency bands, retrieve the phase information. A final algorithm recombines the two channels into a new phase image. The team’s experiments revealed that this method avoids the tendency of recent automatic phase extraction programs to over-represent low frequency signals, a quirk that reduces image resolution. The new reconstruction scheme is especially adept at handling noisy signal inputs, such as low light conditions.

## Introduction

### Phase retrieval: significance and approach overview

The retrieval of the phase of electromagnetic fields is one of the most important and most challenging problems in classical optics. The utility of the phase is that it allows the shape of transparent objects, biological cells in particular, to be quantified in two and three spatial dimensions using visible light^1,2^. In the X-ray band, quantitative phase imaging is also useful because the phase contrast in tissue is orders of magnitude higher than the attenuation contrast^3,4^. The same argument can be made for the identification of liquids^5^ and semiconductor materials for integrated circuit characterization and inspection^6^.

Since only the intensity of a light beam is observable at THz frequencies and above, the phase may be inferred only indirectly from intensity measurements. Computational approaches to this operation may be classified as interferometric/holographic^7,8^, where a reference beam is provided, and noninterferometric, or reference-less, such as direct/iterative^9,10^ and ptychographic^11,12^, which are both nonlinear, and transport-based^13,14^, where the problem is linearized through a hydrodynamic approximation. Direct methods attempt to retrieve the phase from a single raw intensity image, whereas the transport and ptychographic methods implement axial and lateral scanning, respectively. What reference-less methods have in common is the need to obtain intensity measurements at some distance away from the conjugate plane of the object, i.e., with a small defocus. Direct measurement with a defocus is the approach we take here.

All computational phase retrieval approaches, both interferometric and non-interferometric, involve solving a nonlinear and highly ill-posed inverse problem. For direct phase imaging, which is a nonlinear problem—see Section “Formulation of Phase Retrieval as an Inverse Problem”—the classical Gerchberg-Saxton-Fienup (GSF) algorithm^9,10,15^ and its variants^16^ are widely used. They start with a random estimate for the unknown phase distribution and then iteratively update it until the modulus-squared of its Fourier (or Fresnel) transform matches the observed intensity. For well-behaved phase fields, the iteration usually converges to the correct phase^17,18^. Alternatively, the Wiener–Tikhonov functional minimization approach, described in Section “Solution of the Inverse Problem”, exploits prior knowledge about the class of phase objects being imaged to combat noise artefacts.

In 2010, ref. ^19^ proposed a deep neural network in a recurrent scheme to learn the prior from examples as an alternative to using dictionaries^20,21^ as priors. Subsequently, the recursion was unfolded into a cascade for better numerical stability^22^. The physical model of the measurement is taken explicitly into account as a projection operator applied to the reconstruction estimate repeatedly at each recursion or cascade stage. This generalization of dictionaries to deep learning has been successful in a number of linear inverse problems, most notably superresolution^23,24^ and tomography^25,26^.

Recently, deep learning regression has been investigated for application to nonlinear inverse problems, particularly phase retrieval: direct^27–29^, holographic^30,31^, and ptychographic^32,33^. As described briefly in Section “Solution of the Inverse Problem”, a deep neural network (DNN) can be trained in supervised mode from examples of phase objects and their intensity images so that, after training, given an intensity image as input, the DNN outputs an estimate of the phase object. In this case, the physical model is learned implicitly together with the prior from the examples;^27,28^ alternatively, the physical model can be incorporated as a pre-processor^29–31,34,35^, which produces an initial estimate of the phase (the “approximant”) to be used as input to the DNN instead. Extensive reviews of deep learning use for inverse problems can be found in refs. ^26,36,37^.

Here, we propose a new DNN-based computational architecture for phase retrieval with the unique feature of processing low-spatial-frequency and high-spatial-frequency bands as separate channels with two corresponding DNNs trained from an original object database and a high-pass filtered version of the database, respectively. Subsequently, the outputs of the two channels are recombined using a third DNN also specifically trained for this task. The motivation for this new approach is an earlier observation^28^ that nonlinearities in DNN training and execution algorithms tend to amplify imbalances in the spatial frequency content of the training database and in the way different spatial frequencies are treated as they propagate through the physical optical system; this amplified imbalance typically results in lower spatial frequencies becoming dominant and ultimately limiting the resolution of fine spatial features in the reconstructions. A more detailed overview of this phenomenon can be found in Section “Spectral Properties of Training”. Because the essential feature of our newly proposed technique is the synthesis of the two spatial bands through a trained DNN, we refer to it as “learning to synthesize” (LS).

Splitting the spatial frequency content into several bands and processing the bands separately has a long history in signal processing^38–45^. For image reconstruction, dual-band processing has been conducted in fluorescence microscopy^46–48^ and phase retrieval^49^. However, these cases, unlike ours, required structured illumination. In the context of learning-based inversion, the distinction of low and high frequency has been applied to sparse-view CT^50^, based on the theoretical framework of deep convolutional framelets^51^. Moreover, a dual-channel method has been tried for superresolution^52^ (to be understood as upsampling), albeit the two processed channels were combined as a simple convex sum to form the final image. By contrast, the LS method presented here uses a learned nonlinear filter, implemented as a third DNN trained to optimally recombine the two channels according to the spectral properties of the class of objects that the training database represents.

In addition to requiring a single raw image to retrieve the phase through a learned recombination of the spectral channels, the LS method presented here has the desirable property of resilience to noise, especially in the case of weak photon flux down to a single photon per pixel. We achieved this by using an approximant filter^29^ to pre-process the raw image before submitting it to the two spectral channels. The approximant produces an inverse estimate that expressly uses the physical model (a single iteration of the GSF algorithm in ref. ^29^ and here). For very noisy inputs, the approximant is of very poor quality; however, if the subsequent learning architecture is trained with this low-quality estimate as the input, the final reconstruction results are significantly improved. The LS method with the approximant, as presented here, represents a drastic improvement over ref. ^29^, especially in the reconstruction of fine detail, as the latter did not use separate spectral channels to rebalance the frequency content.

### Formulation of phase retrieval as an inverse problem

Let

$${\uppsi}_{{\mathrm{obj}}\left( {x,\,y} \right)} = t\left( {x,y} \right)e^{if\left( {x,y} \right)}$$

denote the complex transmittance of an optically thin object of modulus response *t*(*x*, *y*) and phase response *f*(*x*, *y*), and let ψ_inc_(*x*, *y*) denote the coherent incident field of wavelength *λ* on the object plane. The noiseless intensity measurement *g*_0_(*x*, *y*) (also referred to as a noiseless raw image) is carried out on the detector plane located at a distance *z* away from the object plane and can be written as

1 $$g_{0}(x,y) = \left\vert {\mathbf{F}}_z\left[ {\uppsi}_{\mathrm{inc}}\left(x, y \right) {\uppsi}_{\mathrm{obj}}(x,y) \right] \right\vert^{2} \,{\equiv}\, H_{0}{\mathrm{f}}(x,y)$$

where **F**_*z*_[·] denotes the Fresnel (paraxial) propagation operator for distance *z*, i.e., the convolution

2 $${\mathbf{F}}_z\left[ {\uppsi} \right] = {\uppsi}\left( {x,y} \right) \star \frac{{{\mathrm{e}}^{{\mathrm{i}}2{\mathrm{\pi z}}/{\lambda}}}}{{{\mathrm{i\lambda z}}}}{\mathrm{exp}}\left\{ {i\pi \frac{{x^2 + y^2}}{{{\lambda }}z}} \right\}$$

and *H*_0_ is the (nonlinear) noiseless forward operator. Alternatively, *F*_*z*_ may be expressed in the spatial frequency domain (*v*_*x*_, *v*_*y*_) as

3 $${\mathbf{F}}_z\left[ {\uppsi} \right] = {\cal{F}}^{ - 1}\left\{ {{\cal{F}}\{ {\uppsi}\} {\mathrm{exp}}\left\{ { - i\pi {\uplambda}z\left( {\nu _x^2 + \nu _y^2} \right)} \right\}} \right\}$$

where ${\cal{F}}$ denotes the 2D (spatial) Fourier transform operator and ${\cal{F}}^{ - 1}$ its inverse.

We are interested in weakly absorbing objects, i.e., we assume *t*(*x*, *y*) ≈1. In all the experiments described here, the illumination is also a normally incident plane wave ψ_inc_(*x*, *y*) = 1. Therefore, to a good approximation, we may write

4 $$g_0\left( {x,y} \right) = H_0f\left( {x,y} \right) = \left| {{\mathbf{F}}_{\boldsymbol{z}}\left[ {e^{if\left( {x,\,y} \right)}} \right]} \right|^2$$

This is what we refer to as the direct phase retrieval problem, which Gerchberg–Saxton and related algorithms solve iteratively^9,15^.

In practice, the measurement is subject to Poisson statistics due to the quantum nature of light and to Gaussian thermal noise added by the photoelectric conversion process. We express the noisy measurement as

5 $$g\left( {x,y} \right) = {\cal{P}}\left\{ {p\frac{{H_0f\left( {x,y} \right)}}{{\langle H_0f\rangle}}} \right\} + {\cal{N}}$$

where ${\cal{P}}\{ {\uptheta}\}$ denotes a Poisson random variable with mean θ and ${\cal{N}}$ a Gaussian random variable with zero mean and variance σ^2^. The photon flux in photons per pixel per frame is denoted as *p*, and the spatial average ${\langle H_0f\rangle}= {\langle{g_{0}}\rangle}$ of the noiseless raw image in the denominator is necessary as a normalization factor. The noisy forward operator is *H*, and the purpose of phase retrieval is to invert *H* to recover *f* as accurately as possible, despite the nonlinearity and randomness present in the measurements.

### Solution of the inverse problem

The Wiener–Tikhonov approach to solving inverse problems of the form *g* = *Hf* is to obtain the estimate $\hat f$ of the inverse as

6 $$\widehat f = {\mathrm{argmin}}_f\left\{ {D\left( {H_0f\!,\,g} \right) + {\mathrm{\Phi }}\left( f \right)} \right\}$$

Here, *D*(*H*_0_*f*, *g*) is the fitness term (or data-fidelity term), where *D*(·,·), is a distance operator that should be determined based on the statistics o the noise involved. When machine learning is used to approximate (Eq. (6)), the dilemma of choosing the proper distance operaor shifts to choosing the loss function for training a deep neural network^53^. We address this latter problem in some detail in Section “Design and Training of the DNNs in the LS-DNN”.

The second term Φ(*f*) in Eq. (6) is the regularizer, or prior knowledge term. Its purpose is to compete with the fitness term in the minimization to mitigate ill-posedness in the solution. That is, the regularizer penalizes solutions that are promoted by the noise in the forward problem, as in Eq. (5) for example, but does not meet general criteria known a priori for valid objects.

The prior may be defined explicitly, e.g., as a minimum energy^54^ or sparsity^55–59^ criterion, or learned from examples as a dictionary^20,21,60,61^ or through a deep learning scheme^19,22,24–33^.

Here, as in earlier works on direct phase retrieval^27–33^, and due to the nonlinearity of the forward model, we adopt the end-to-end and approximant methods. These we denote as

7 $${\mathrm{End}} - {\mathrm{to}} - {\mathrm{End}}:\hat f = {\mathrm{DNN}}\left( g \right)\quad {\mathrm{and}}$$

8 $${\mathrm{Approximant}}:\hat f = {\mathrm{DNN}}\left( {\hat {f^ \ast }} \right)$$

where DNN(.) is the output of a deep neural network and ${\hat f}^ \ast$ is the approximant, which we will describe shortly. In the end-to-end approach, the burden is on the DNN to learn from examples both the forward operator *H*_0_ and the prior Φ to execute, in one shot, an approximation to the ideal solution (Eq. (6)). Training takes place in supervised mode, with known pairs of phase objects *f* and their raw intensity images *g* generated on a phase spatial light modulator (SLM) and measured on a digital camera, respectively. Note that training is generally slow, taking several hours if a few thousand examples are used. However, after training is complete, the execution of Eq. (7) or Eq. (8) is very fast, as it requires only forward (non-iterative) computations. This is one significant advantage over the standard way of minimizing the Wiener–Tikhonov functional (Eq. (6)) iteratively for each image.

When the inverse problem becomes severely ill-posed or the noise is extremely strong, the learning burden on the DNN becomes too high; then, generally, better results are obtained by training the DNN to receive as input the approximant ${\hat f}^ \ast$ instead of the raw measurement *g* directly. The approximant is obtained through an approximate inversion of the forward operator; for example, in ref. ^30^, it was implemented as a digital holographic backpropagation algorithm, whereas in ref. ^29^, it was the outcome of a single iteration of the Gerchberg–Saxton algorithm^9^. While these approximants ${\hat f}^ \ast$ generally do not look very good, especially in highly noisy situations^29^, through training, the DNN is able to learn a better association of ${\hat f}^\ast$ with its corresponding true object *f* than what it can learn with the noisy raw measurement *g*.

### Spectral properties of training

The design of deep neural networks is an active field of research, and a comprehensive review of methods and caveats is well beyond the scope of this paper. We refer the reader to refs. ^26,36,37^ for more extensive background and references. Here, we discuss the influence on the quality of training of the spatial power spectral density of the database from which examples are drawn.

In both the end-to-end and approximant methods (Eqs. 7–8), the training examples determine the object class prior to be learned by the DNN. In ref. ^28^, we addressed the influence of the spatial power spectral density (PSD) *S*(*v*_*x*_, *v*_*y*_) of the example database on the quality of training. It is well known^62–66^ that two-dimensional (2D) images of natural objects, such as those contained in ImageNet^67^, follow the inverse quadratic PSD law

9 $$S\left( {\nu _x,\nu _y} \right) = \frac{1}{{\nu _x^2 + \nu _y^2}}$$

Other types of object classes of practical interest exhibit similar power-law decay, perhaps with slightly different exponents. This observation means that if a neural network is trained on such an object class, higher spatial frequencies are presented less frequently to the DNN during the training stage. At face value, this scenario is as it should be, since the relative popularity of different spatial frequencies in the database is precisely one of the priors that the DNN ideally should learn.

This understanding needs to be modified in the context of inverse problems because the representation of high spatial frequencies in the raw images is also uneven—typically to the disadvantage of the high spatial frequencies. In the specific case of phase retrieval, higher spatial frequencies within the spatial bandwidth (as determined by the numerical aperture NA) have a uniform transmission modulus but are more severely scrambled by the chirped oscillations of the transfer function (Eq. (3)). Thus, higher spatial frequencies suffer a double penalty:^28^ their recovery becomes more sensitive to noise due to scrambling, and they are less popular due to the inverse-square (or similar) PSD law; thus, they are presented less frequently than their fair share to the DNN training process. Moreover, since the DNN itself and its training routine are both highly nonlinear, there is an acute risk that any unevenness in the treatment of different spatial frequency bands may be amplified in the final result, eventually causing the lower frequencies to dominate.

In ref. ^28^, the authors attributed the inability of the phase extraction neural network (PhENN)^27^ to resolve spatial features well within its admitted spatial bandwidth to this unequal treatment of spatial frequencies. They showed that the resolution of PhENN is approximately doubled by pre-filtering the training examples to flatten their PSD. That is, during the training, each example *f*(*x*, *y*) from the database was replaced with its filtered version

10 $$f_p\left( {x,y} \right): = {\cal{F}}^{ - 1}\left\{ {{\cal{F}}\left\{ {f\left( {x,y} \right)} \right\} \times C\left( {\nu _x,\nu _y} \right)} \right\}$$

The transfer function was defined as the high-pass filter

11 $$C\left( {\nu _x,\nu _y} \right) = \sqrt {\nu _x^2 + \nu _y^2}$$

exactly compensating for the inverse-quadratic dependence (Eq. (9)) and flattening the spectrum. The raw images for training were correspondingly filtered as

$$g_p\left( {x,y} \right) = Hf_p\left( {x,y} \right)$$

whereas, during the test, the un-filtered measurements (i.e., as received from the camera) were used to obtain the reconstructions. Unfortunately, with this implementation, amplification of high-spatial-frequency features, especially of artefacts caused even by weak noise, was also evident in the reconstructions. This outcome is not surprising since, technically, (Eq. (10)) trades off violating the prior for a finer spatial resolution. The LS approach that we describe next is meant to fix this problem.

### The LS scheme: spectral band-specific training and operation

**Fig. 1:**
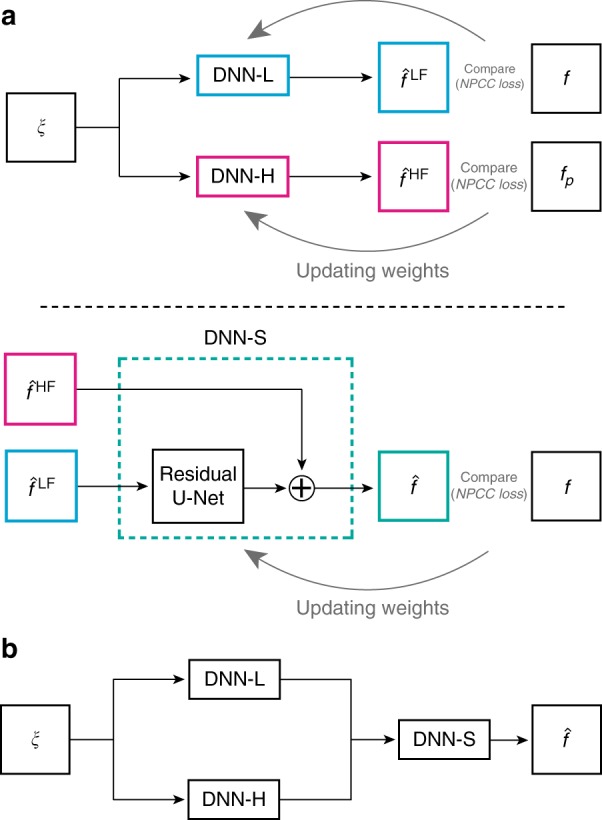
LS-DNN schematic. **a** Training stage. **b** Test stage

Motivated by the spectral-domain observations described earlier, we construct the LS block diagram in Fig. 1 to process low and high spatial frequencies separately and then synthesize them. In the final estimate, the high-frequency components are restored without significant artefacts, even in the presence of strong noise. Here, ξ is the input to the LS system, i.e*.*, the intensity in the end-to-end scheme or an initial estimate of the unknown phase produced by the pre-processor in the approximant scheme.

The LS system itself consists of three deep neural networks, which we denote as DNN-L, H, and S. DNN-L is trained with unfiltered examples, and its output ${\hat f}^{\mathrm{LF}}$ generally behaves well at low spatial frequencies but misses fine details in the reconstructions. DNN-H is trained to produce high-pass filtered outputs ${\hat f}^{\mathrm{HF}}$ of unfiltered inputs; thus, it performs the crucial function of preserving the upper end of the spectrum. The filter function is chosen according to (Eq. (11)), but more generally as

12 $$C\left( {\nu _x,\nu _y} \right) = \left( {\nu _x^2 + \nu _y^2} \right)^q$$

The power law *q* and its influence on reconstruction quality are investigated in detail below.

We train DNN-S so that from the outputs ${\hat f}^{\mathrm{LF}}$, ${\hat f}^{\mathrm{HF}}$ of DNN-L and H, respectively, DNN-S can synthesize a final image $\hat f$ with good behavior at all spatial frequencies. The details of how the three networks are structured, trained and operated according to the LS scheme are in presented Section “LS scheme Implementation, Training, and Operation”.

## Results

**Fig. 2:**
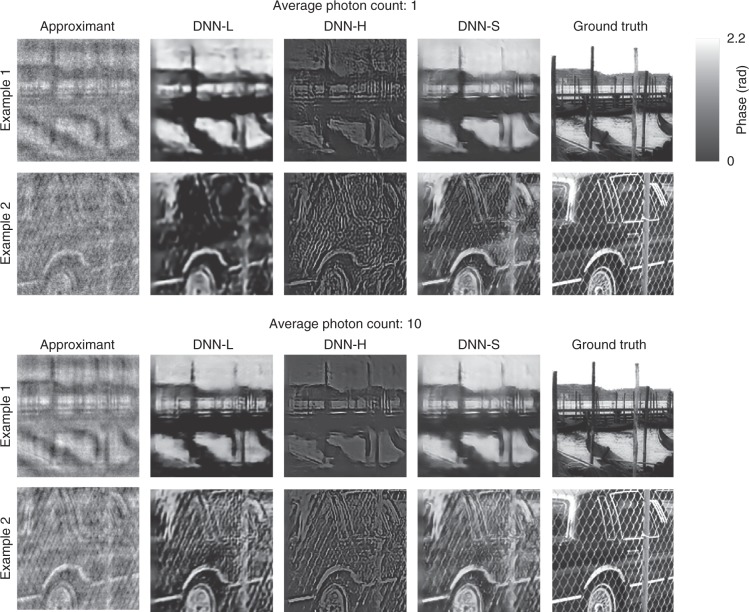
Reconstructions by LS-DNN. Top: 1 photon/pixel/frame, bottom: 10 photons/pixel/frame; from left to right: approximant (the input to the LS-DNN system), DNN-L reconstruction^29^, DNN-H reconstruction (*q* = 0.5), DNN-S reconstruction, and ground truth

Figure 2 shows the reconstructions obtained by the LS-DNN method (*q* = 0.5) and its components at fluxes *p* = 1 photon and 10 photons per pixel, as defined immediately above. As expected, the reconstructions ${\hat f}^{\mathrm{LF}}$ by DNN-L have good fidelity at low spatial frequencies but lose fine details, as in ref. ^29^, whereas the reconstructions ${\hat f}^{\mathrm{HF}}$ by DNN-H appear to be high-pass filtered versions of the true objects with some additional high-frequency artefacts due to the noise. The reconstructions $\hat f$ by DNN-S preserve detail at both low and high frequencies while significantly attenuating the artefacts. The improvement in $\hat f$over ${\hat f}^{\mathrm{HF}}$is more pronounced under severe noise, i.e., in the *p* = 1 photon/pixel case. More examples of reconstructions (obtained with *q* = 0.5) for the noisier case (*p* = 1) are given in the Supplementary Material.

**Fig. 3:**
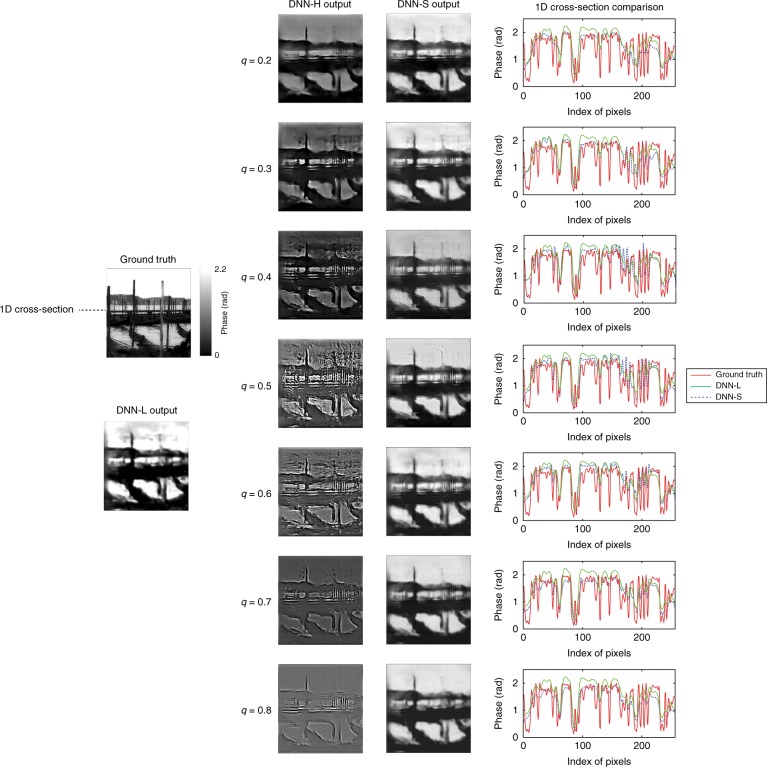
Comparisons of LS-DNN reconstructions under different *q* values for *p* = 1 photon/pixel. Columns from left to right: ground truth and DNN-L output; DNN-H output under different *q* values; DNN-S output under different *q* values; and 1D cross-section (along the dashed line indicated in the ground-truth image) of (i) DNN-L output (green), (ii) DNN-S output under different *q* values (blue dashed) and (iii) ground truth (red)

**Fig. 4:**
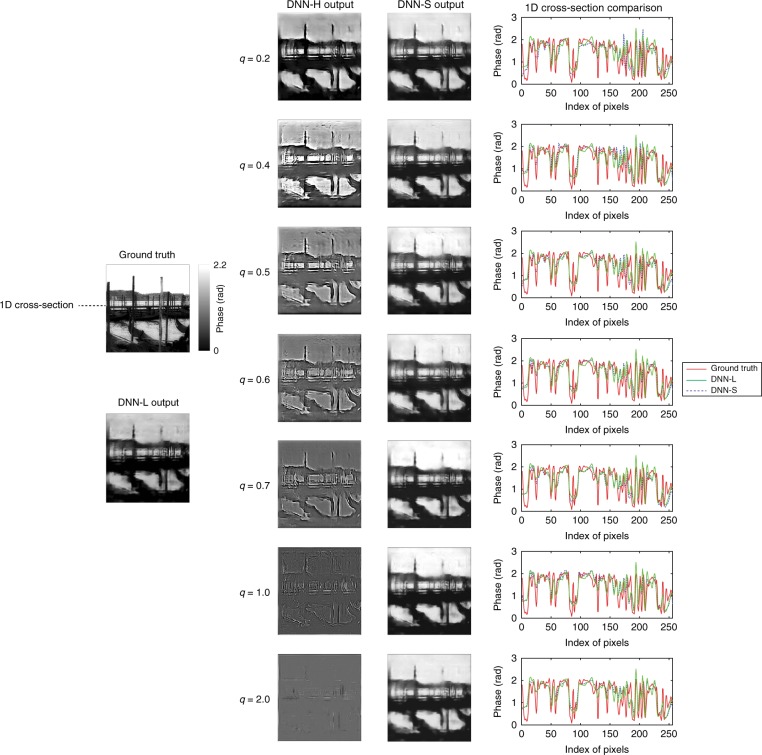
Comparisons of LS-DNN reconstructions under different *q* values for *p* = 10 photons/pixel. Columns from left to right: ground truth and DNN-L output; DNN-H output under different *q* values; DNN-S output under different *q* values; and 1D cross-section (along the dashed line indicated in the ground-truth image) of (i) DNN-L output (green), (ii) DNN-S output under different *q* values (blue dashed) and (iii) ground truth (red)

In Figs. 3 and 4, we compare reconstructions by LS-DNN with different values of the pre-filtering parameter *q* for *p* = 1 photon and *p* = 10 photons per pixel, respectively. The most detail at high frequencies in the DNN-S output is preserved in the range $0.3\, \lesssim\, q\, \lesssim\, 0.7$. At lower values of *q*, the quality of the reconstructions by DNN-S does not noticeably exceed that of DNN-L. This result is expected, since in the limit *q* = 0, training DNN-H becomes identical to training DNN-L. On the other hand, for values $q \gtrsim 0.7$, the DNN-H output is dominated by high-frequency artefacts, and again, the quality of DNN-S reconstructions regresses to that of DNN-L, since the high-frequency channel is no longer contributing. These observations are valid for both values of *p* and even stronger for the most severely noise-limited case *p* = 1.

Similar trends are evident according to various quantitative metrics averaged over the entire set of test examples compared to the true phase signals *f*, summarized in Table 1. For comparison, we used the peak signal-to-noise ratio (PSNR)^68^, the structural similarity index metric (SSIM)^69,70^, and the Pearson correlation coefficient (PCC), defined as^71,72^:

13 $${\mathrm{PCC}}\left( {a,b} \right) \equiv \frac{{\mathop {\sum}\limits_{x,y} {\left( {a\left( {x,y} \right) - \langle a\rangle } \right)\left( {b\left( {x,y} \right) -\langle b\rangle} \right)} }}{{\sqrt {\mathop {\sum}\limits_{x,y} {\left( {a\left( {x,y} \right) - \langle a\rangle } \right)^2} } \sqrt{\mathop {\sum}\limits_{x,y} {\left( {b\left( {x,y} \right) - \langle b\rangle } \right)^2}} }}$$

where ${\langle{a}\rangle}$ and ${\langle{b}\rangle}$ are the spatial averages of the generic functions *a*(*x*, *y*), *b*(*x*, *y*). If *a* and *b* are uncorrelated, PCC(*a*, *b*) is zero, whereas if they are identical, then PCC(*a*, *b*) = 1. More quantitative comparisons, including the comparison of DNN-S and DNN-L reconstructions for all 500 test images and comparisons with alternative quantitative metrics, i.e*.*, the root mean square error and peak-to-valley error, are available in Sections 4 and 10 of the Supplementary Material, respectively. (Since DNN-H is trained with a spectrally pre-filtered version of the true object *f*, quantitative comparison of its output with the ground truth does not make sense.)

**Table 1:** Quantitative comparison of reconstructions by approximant, DNN-L and DNN-S based on a test set of 500 images. Each entry takes the form ’average ± standard deviation’.

	Average PCC ± std.dev		Average PSNR ± std.dev (dB)		Average SSIM ± std.dev	
	*p* = 1	*p* = 10	*p* = 1	*p* = 10	*p* = 1	*p* = 10
Approximant *f*^ˆ∗^	0.148 ± 0.070	0.182 ± 0.086	8.448 ± 4.182	8.465 ± 4.190	0.231 ± 0.111	0.233 ± 0.112
DNN-L output *f*^ˆLF^	0.812 ± 0.126	0.878 ± 0.083	16.520 ± 2.693	18.439 ± 2.811	0.878 ± 0.088	0.923 ± 0.063
DNN-S output *f*^ˆ^ (*q* = 0.1)	0.847 ± 0.111	0.891 ± 0.078	17.596 ± 2.612	18.653 ± 2.289	0.903 ± 0.078	0.928 ± 0.065
DNN-S output *f*^ˆ^ (*q* = 0.2)	0.857 ± 0.088	0.895 ± 0.079	17.816 ± 2.821	18.716 ± 2.286	0.906 ± 0.063	0.928 ± 0.058
DNN-S output *f*^ˆ^ (*q* = 0.3)	0.859 ± 0.105	0.896 ± 0.074	18.017 ± 2.583	18.749 ± 2.228	0.910 ± 0.075	0.932 ± 0.065
DNN-S output *f*^ˆ^ (*q* = 0.4)	0.865 ± 0.104	0.895 ± 0.073	18.234 ± 2.484	19.040 ± 2.284	0.926 ± 0.069	0.934 ± 0.057
DNN-S output *f*^ˆ^ (*q* = 0.5)	0.869 ± 0.112	0.897 ± 0.073	18.600 ± 2.297	19.072 ± 2.271	0.929 ± 0.081	0.935 ± 0.056
DNN-S output *f*^ˆ^ (*q* = 0.6)	0.869 ± 0.108	0.898 ± 0.077	18.566 ± 2.540	19.041 ± 2.264	0.926 ± 0.080	0.935 ± 0.060
DNN-S output *f*^ˆ^ (*q* = 0.7)	0.864 ± 0.125	0.895 ± 0.079	17.827 ± 2.377	19.032 ± 2.267	0.927 ± 0.086	0.932 ± 0.069
DNN-S output *f*^ˆ^ (*q* = 0.8)	0.845 ± 0.115	0.893 ± 0.076	17.577 ± 2.546	19.031 ± 2.755	0.902 ± 0.081	0.931 ± 0.063
DNN-S output *f*^ˆ^ (*q* = 1)	0.821 ± 0.147	0.890 ± 0.078	17.051 ± 2.306	18.841 ± 2.717	0.902 ± 0.092	0.931 ± 0.063
DNN-S output *f*^ˆ^ (*q* = 2)	0.819 ± 0.113	0.882 ± 0.078	16.822 ± 2.586	18.645 ± 2.860	0.889 ± 0.081	0.928 ± 0.059

It is noteworthy that in both visualization and quantitative comparisons of Figs. 3, 4 and Table 1, respectively, the performance of DNN-S remains approximately the same within the range $0.3\, \lesssim\, q\, \lesssim \,0.7$. This is desirable, as it suggests that one need not pre-filter exactly with the inverse of the PSD power law. This further suggests that for datasets that do not represent natural images and may obey power laws different from (Eq. (9)), not knowing the exact value of *q* may not be catastrophic. We have not tested this hypothesis exhaustively, as it is beyond the scope of this paper.

In Table 2 and in the Supplementary Material, we also analyze the case of a larger DNN (denoted as DNN-L-3) with computational capacity equal to the sum of DNN-L, H and S, though trained with un-filtered examples, and show that DNN-L-3 cannot achieve reconstructions of even quality. Therefore, the improvements over ref. ^29^ resulted from the training procedure followed in the LS-DNN method and did not simply occur by brute force due to the use of a larger computational capacity.

**Table 2:** Quantitative comparison of reconstructions by approximant, DNN-L-3 and DNN-S (for *q* = 0.5) based on a test set of 500 images. Each entry takes the form’average ± standard deviation’.

	Average PCC ± std.dev		Average PSNR ± std.dev (dB)		Average SSIM ± std.dev	
	*p* = 1	*p* = 10	*p* = 1	*p* = 10	*p* = 1	*p* = 10
Approximant *f*^ˆ∗^	0.148 ± 0.070	0.182 ± 0.086	8.448 ± 4.182	8.465 ± 4.190	0.231 ± 0.111	0.233 ± 0.112
DNN-L output *f*^ˆLF^	0.812 ± 0.126	0.878 ± 0.083	16.520 ± 2.693	18.439 ± 2.811	0.878 ± 0.088	0.923 ± 0.063
DNN-L-3 output *f*^ˆL-3^	0.811 ± 0.154	0.879 ± 0.107	16.529 ± 2.549	18.368 ± 2.322	0.875 ± 0.086	0.926 ± 0.094
DNN-S output *f*^ˆ^ (*q* = 0.5)	0.869 ± 0.112	0.897 ± 0.073	18.600 ± 2.297	19.072 ± 2.271	0.929 ± 0.081	0.935 ± 0.056

**Fig. 5:**
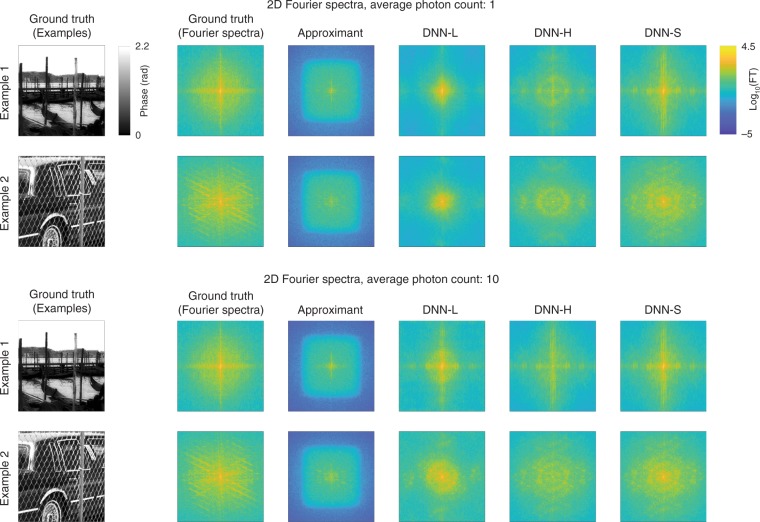
Fourier spectra of two test examples and their reconstructions from the components of the LS scheme. Top: 1 photon/pixel. Below: 10 photons/pixel

**Fig. 6:**
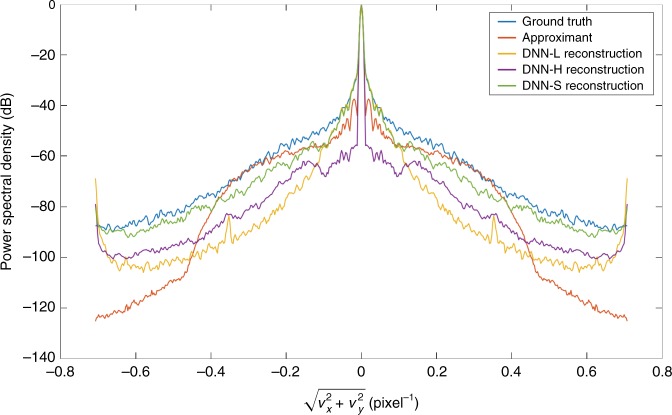
1D diagonal cross-sections of the average 2D power spectral density (PSD) of test set images for *p* = 1 photon per pixel

To further study the behavior of the LS components in the low-spatial-frequency and high-spatial-frequency bands, we studied the reconstructions in the Fourier domain. Figure 5 shows the spectra (2D Fourier transforms) of two randomly selected test examples. Figure 6 and Fig. S5 in the Supplementary Material show normalized diagonal cross-sections of the PSD averaged over all test images for *p* = 1 and 10 photons per pixel, respectively. These plots illustrate that the outputs of DNN-L and DNN-H are depleted at high and low frequencies, respectively, with the losses being more severe in the noisy case *p* = 1, whereas the output of DNN-S mostly recovers the frequency content at both bands, albeit still with some minor loss at high frequencies.

**Fig. 7:**
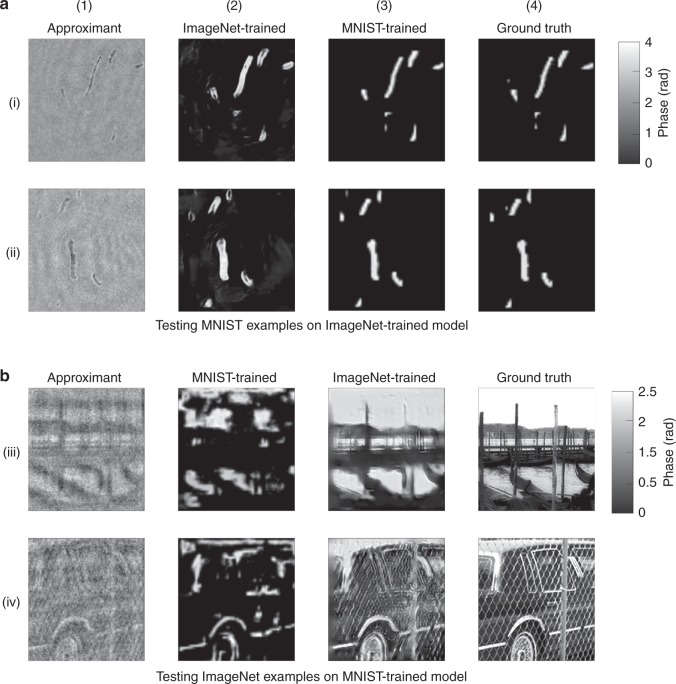
Cross-domain generalization of LS-DNN. (**a**) predicting MNIST examples by ImageNet-trained LS model; (**b**) predicting ImageNet examples by MNIST-trained LS model

Often, access to a large number of annotated training examples that are in the exact same class as that of the test examples is not possible. Therefore, it would be desirable if a deep learning algorithm trained on a standard dataset could generalize reasonably well even if tested directly on a different dataset. To evaluate the cross-domain generalization ability of LS, we take two representative datasets: ImageNet and MNIST. ImageNet is a dataset that offers broad prior information and thus weaker regularization to the training when used as the training set, whereas MNIST is a dataset that offers constrained prior information and thus stronger regularization to the training when used as the training set. In Fig. 7a, we see that if the LS model is trained with ImageNet, as we have done in this paper, predicting examples from a completely different MNIST dataset offers a similar performance to that when the training is done on MNIST; however, if the model is trained on a more constrained MNIST dataset, the performance when predicting ImageNet examples is poor, and the reconstructions display sparse features resembling the MNIST examples, most obviously in Fig. 7 row (iv), column (2). This is an indication of the unduly strong regularization effect that the MNIST examples impose on the training process and verifies our choice of training the LS with the more general dataset, i.e., ImageNet, which is beneficial for the model’s generalization ability. The quantitative comparison, available in Section 12 and Table S6 of the Supplementary Material, also supports our claim above. In general, DNNs trained on more general datasets, e.g., ImageNet, typically generalize well to more constrained datasets, e.g., MNIST, whereas the opposite is not generally true^27,37,73^.

Last, we experimentally characterized the spatial resolution of the LS-DNN reconstructions, i.e*.*, the ability of DNN-S to resolve two pixels at nearby locations having a phase delay higher than the rest of the signal. Similar analyses were carried out in refs. ^28,73^, where the methodology was also described in detail. In the work presented here, we carried out the analysis under ample illumination, i.e*.*, not under strong Poisson statistics. We made that choice because spatial resolution under highly noisy conditions becomes non-trivially coupled to the noise statistics, and a complete investigation would have been outside the scope of the present investigation. The results, demonstrating an improved spatial resolution of LS-DNN reconstructions over ref. ^27^, are shown in Section 6 of the Supplementary Material.

## Discussion

The LS-DNN reconstruction scheme for quantitative phase retrieval has been shown to be resilient to highly noisy raw intensity inputs while preserving high-spatial-frequency details better than those of ref. ^29^. Moreover, the robustness of the reconstructions to variations in the pre-filtering power law *q* of ≈1/2 following from natural image statistics and the good generalization ability of LS to other classes of objects make the approach efficient and practical.

Beyond the scope of the work reported here, further improvements may be obtained through modifying the architecture of the DNNs used to process and recombine the two spatial frequency bands. Another obvious alternative strategy is to split the signals into more than two bands and then process and recombine these multiple bands with a synthesizer DNN according to the LS scheme. While we did not investigate this approach in detail here, we expect it to present a trade-off between the improvements and the complexity of having to train multiple neural networks, implying the need for more examples and the danger of poor generalization.

## Materials and methods

### LS scheme implementation, training, and operation

Here, we describe in full detail the LS scheme of Fig. 1. Attempts at solving noiseless inverse problems by a similar method can be found in our earlier work^74^. For unity in notation, we denote the input to the entire LS system as ξ(*x*, *y*), to be understood as the intensity pattern in the end-to-end scheme and the approximant in the approximant scheme.

We discuss the approximant implementation in more detail in Section “Computation of the Approximant”.

The two training steps are shown in block-diagram form in Fig. 1. The first step consists of training two separate DNNs in parallel, as follows:

- DNN-L is trained to match unfiltered patterns ξ^(*n*)^(*x*, *y*) at its input with the corresponding unfiltered example phase patterns *f*^(*n*)^(*x*, *y*) as the ground truth at its output (the superscript *n* enumerates the examples).
- DNN-H is trained to match unfiltered patterns ξ^(*n*)^(*x*, *y*) at its input to the corresponding spectrally filtered (according to (12)) versions $f_P^{\left( n \right)}\left( {x,y} \right)$ of the ground-truth examples *f*^(*n*)^(*x*, *y*) at its output.

The output of DNN-L for a general test input ξ(*x*, *y*) is denoted as ${\hat f}^{\mathrm{LF}}\left( {x,y} \right)$. Assuming similar training conditions, ${\hat f}^{\mathrm{LF}}$ matches the output of the PhENN as presented in ref. ^27^ in the end-to-end scheme or ref. ^29^ in the approximant scheme; that is, ${\hat f}^{\mathrm{LF}}$ is expected to be fairly accurate at low spatial frequencies but without fine details.

The output of DNN-H is denoted as ${\hat f}^{\mathrm{HF}}\left( {x,y} \right)$. Note that ref. ^28^ required spatial pre-filtering of the raw inputs *g*; here, we do not spatially pre-filter the input ξ (i.e., *g* or ${\hat f} ^\ast$according to whether the end-to-end or approximant scheme is used). We instead train DNN-H to produce the filtered output based on an unfiltered input. This leads to better generalization because DNN-H is trained on the broadest set of possible images (whereas the training in ref. ^28^ was on high-frequency images only). Moreover, using unfiltered inputs for DNN-H allows the training process to be parallelized for better efficiency.

Depending on the value of the power law *q* in Eq. (12), the PSD of the patterns used to train DNN-H will be flat or almost flat. The output of DNN-H ${\hat f}^{\mathrm{HF}}\left( {x,y} \right)$ is expected to have better fidelity at fine spatial features of the phase objects. However, spectral flattening may also generate artefacts due to overlearning high spatial frequencies. Therefore, ${\hat f}^{\mathrm{HF}}$ looks rather like a high-pass-filtered version of the true object *f*, which we found to be more beneficial for subsequent use in the LS scheme.

The second training step consists of combining the two partially accurate reconstructions ${\hat f}^{\mathrm{LF}}$ and ${\hat f}^{\mathrm{HF}}$ into a final estimate $\hat f\left( {x,y} \right)$ with uniform quality at all spatial frequencies, low and high, up to the passband. To this end, we train the synthesizer DNN-S to receive ${\hat f}^{\mathrm{LF}}$ and ${\hat f}^{\mathrm{HF}}$ as inputs and use the unfiltered examples *f* as the output. To avoid any further damage to the high-spatial frequency content in ${\hat f}^{\mathrm{HF}}$, we bypass ${\hat f}^{\mathrm{HF}}$ and present it intact to the last layer of DNN-S. By operating on ${\hat f}^{\mathrm{HF}}$ alone, DNN-S learns how to treat the low-frequency reconstruction to compensate for artefacts at all bands. The use of the synthesizer DNN-S also makes our results less sensitive to the choice of power *q* in the transfer function (Eq. (12)). We found that $q \in \left[ {0.3,0.7} \right]$ can produce reconstructions of approximately even quality, as presented in Section “Results”.

After DNN-L, DNN-H, and DNN-S have been trained, they are combined in the LS system and operated as shown in Fig. 1b. The input ξ(*x*, *y*) is passed to DNN-L and DNN-H in parallel fashion, and the respective outputs ${\hat f}^{\mathrm{LF}}\left( {x,y} \right)$ and ${\hat f}^{\mathrm{HF}}\left( {x,y} \right)$are passed to DNN-S, which produces the final estimate $\hat f\left( {x,y} \right)$. It is worth noting that it is *not* valid to lump the three networks in Fig. 1b into a single network, due to their separate training schemes described above.

### Experimental apparatus and data acquisition

In each experiment carried out to train and test different LS-DNN schemes, 10,450 image objects from ImageNet^67^ were successively projected on a phase SLM as phase objects (i.e., with a phase value at each pixel proportional to the intensity of the corresponding pixel in the original from the database), and their raw images were recorded by an EM-CCD camera at an out-of-focus plane. More information on the SLM used is available in Section 8 of the Supplementary Material. These 10,450 ground-truth phase images and their corresponding raw intensity images were split into a training set of 9,500 images, a validation set of 450 images and a test set of 500 images. The choice of ImageNet^67^ is reasonable, since the low-frequency dominance in its spatial PSD is representative of the broader classes of objects of interest, and therefore, we anticipate that our results will generalize well in practical applications.

**Fig. 8:**
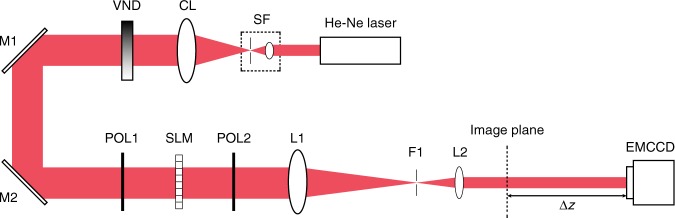
Optical apparatus for experiments. SF: spatial filter, CL: collimating lens, VND: variable neutral density filter

The experiments were carried out using the apparatus described in Fig. 8. The light source was a continuous wave helium-neon gas laser at 632.8 nm. The laser beam first passed through a variable neutral density filter (VND) that served the purpose of adjusting the photon flux. The beam was then spatially filtered and expanded into an 18 mm diameter collimated pencil and sent onto a transmission SLM of 256 × 256, each of size 36 × 36 μm. Phase objects were projected onto the SLM and imaged by a telescope (4F system) consisting of lenses L1 (focal length 230 mm) and L2 (100 mm). The 2.3× reduction factor in the 4F system was designed to reduce the spatial extent of the defocused raw image to approximately fit the size of the camera. An aperture was placed in the Fourier plane to suppress higher diffraction orders due to the periodicity of the SLM pixels. The raw intensity images were captured by a Q-Imaging EM-CCD camera with 1004 × 1002 square-shaped pixels of size 8 × 8 μm placed at a distance *z* = 400 mm from the image plane of the 4F system. Additional details about the implementation of the optical apparatus and its numerical simulation with digital Fresnel transforms are provided in the Supplementary Material.

The photon flux is quantified as the number of photons *p* received by each pixel on average for an unmodulated beam, i.e., with no phase modulation driving the SLM. During an initial calibration procedure, for different positions of the VND filter, the photon level is measured using a calibrated silicon photodetector placed at the position of the camera. The quoted photon count *p* is also corrected for the quantum efficiency of the CCD (60% at λ = 632.8 nm), meaning that we refer to the number of photons actually detected and not the incident number of photons.

Here, we report results for two levels of photon flux *p* = 9.8 ± 5% and 1.1 ± 5%, quoted in the text as “10” and “1” photons, respectively. The data acquisition, training and testing procedures of the entire LS-DNN architecture were repeated separately for each value of *p*. For each photon count, the acquisition of all intensity images takes approximately 50 min, and the computation of all approximants takes approximately 2.9 hours using MATLAB on a regular CPU (or equivalently, approximately 1 s per example).

### Design and training of the DNNs in the LS-DNN

There is a wide variety of DNN structures one may choose to implement DNN-L, H and S. In this work, we use the same architecture as in ref. ^29^ for DNN-L, i.e., a residual U-net architecture with skip connections^75^. For simplicity, DNN-H and DNN-S are also chosen to be structures similar to DNN-L. The details of the implementations, the training curves, and the validation loss when less training data were used are given in Section 1 and Section 9, respectively, of the Supplementary Material. We made these choices of architectures and training specifics to enable fair comparisons with the earlier works; alternative architectures are certainly possible within the LS scheme, though we judged a full exploration to be outside the scope of the present paper.

The training of a neural network is typically implemented as a stochastic optimization procedure^76,77^, where the neural network internal coefficients (weights) are adjusted to minimize a metric of the distance between the actual output of the neural network and its desired output to a given input (training example). This distance is called the training loss function (TLF). In the context of training to solve an inverse problem, the TLF is defined as

14 $${\cal{L}}={\sum \nolimits_n}D\left({\hat f}^{(n)},f^{(n)}\right)$$

where the superscript n is again used to enumerate the examples and the dilemma of choosing the appropriate metric operator *D* emerges.

It is generally accepted^27,78–80^ that the L^2^ metric (also referred to as the mean square error, MSE) is a poor choice that does not generalize well, i.e., deep neural networks trained with the MSE do not perform well when presented with examples outside their training set. For image classification tasks, and in an early work on phase retrieval^27^, the *L*^1^ metric (mean absolute error, MAE) was used instead. In direct analogy with compressive sensing, the *L*^1^ metric promotes sparsity *in the internal connectivity* of the neural network, which leads to better generalization. However, ref. ^73^ found that in highly ill-posed problems, this benefit is eclipsed by the inability of the MAE and pixel-wise metrics more generally to learn spatial structure priors about the object class that are crucial for regularization.

In this paper, we train DNN-L, H, and S using the negative Pearson correlation coefficient (NPCC)^29,73^ as the TLF. The NPCC is defined as in Eq. (13) but with a negative sign. Thus, training the neural network minimizes the TLF towards ${\cal{L}} \approx - N$, where *N* is the number of training examples.

The NPCC has been shown^81^ to be more effective in recovering fine features than conventional loss functions such as the mean square error (MSE), mean absolute error (MAE) and structural similarity (SSIM) index^69,70^. However, the NPCC is invariant to affine transformations to its arguments, i.e*.*,

15 $$D_{{\mathrm{NPCC}}}\left( {a,b} \right) = D_{{\mathrm{NPCC}}}\left( {{\upalpha}_1a + {\upalpha}_2,{\upbeta}_1b + {\upbeta}_2} \right)$$

for arbitrary real numbers α_1_, α_2_, β_1_, β_2_. For *quantitative* phase retrieval, where the scale of the phase difference matters, the affine ambiguity is resolved with a histogram equalization step after inversion^28^.

### Computation of the approximant

It has been shown that even under extreme noise conditions, just a single iteration of the Gerchberg–Saxton (GS) algorithm suffices as an approximant in scheme (Eq. (8)) for phase retrieval^29^. We elected to use the same approach here for the LS-DNN architecture. More recently, a comparative study^82^ showed that higher iterates or regularized versions of GS do improve the appearance of the approximant result ${\hat f^\ast}$ but do not yield a significant improvement in the end output $\hat f$ of the DNN. Similar conclusions hold for alternatives to GS, e.g*.*, gradient descent. While these alternative schemes are interesting for the LS-DNN method, we chose to not pursue them here.

The general form of the (*k* + 1)-th GS iterate from the *k*-th iterate is

16 $$f\left[ {k + 1} \right] = arg\left\{ {{\mathbf{F}}_z^{ - 1}\left( {\sqrt g \,{\mathrm{exp}}\left\{ {i\,arg\left\{ {{\mathbf{F}}_z\left( {e^{jf[k]}} \right)} \right\}} \right\}} \right)} \right\}$$

where we have taken into account that ψ_inc_ = 1. Accordingly, our approximant is

17 $${\hat f^\ast} = f\left[ 1 \right] = arg\left\{ {{\mathbf{F}}_z^{ - 1}\left( {\sqrt g \,{\mathrm{exp}}\,\left\{ {i\,arg\left\{ {{\mathbf{F}}_z\left( 1 \right)} \right\}} \right\}} \right)} \right\}$$

where **1** denotes the function that is uniformly equal to one within the frame^82^.

**Fig. 9:**
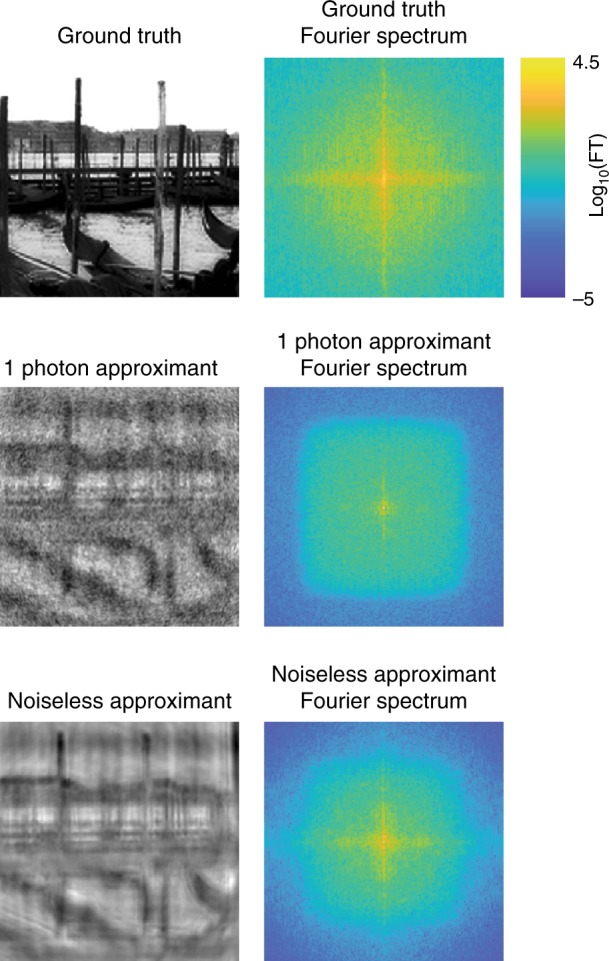
2D Fourier spectrum comparison. From top to bottom: ground-truth; noiseless approximant; and approximant for 1 photon/pixel illumination

Figure 9 compares the 2D (log-scale) Fourier spectrum magnitude of a ground-truth image (from ImageNet^67^), the approximant (Eq. (16)) computed without noise, and the approximant (Eq. (16)) computed from an input subject to Poisson statistics corresponding to an average flux of one photon per pixel. We can see that although the single-photon approximant (which we used as the input for the LS-DNN) has a large support in its spectrum, it is the noise that dominates the mid-to-high frequency range. Therefore, the learning process still bears the burden of restoring the correct high-frequency contents, and relying heavily on high-frequency priors, as our DNN-H does, is justified.

## Supplementary information

Supplementary Information

## References

[1] Marquet P (2005). Digital holographic microscopy: a noninvasive contrast imaging technique allowing quantitative visualization of living cells with subwavelength axial accuracy. Opt. Lett..

[2] Popescu G (2006). Diffraction phase microscopy for quantifying cell structure and dynamics. Opt. Lett..

[3] Mayo SC (2003). X-ray phase-contrast microscopy and microtomography. Opt. Express.

[4] Pfeiffer F (2006). Phase retrieval and differential phase-contrast imaging with low-brilliance X-ray sources. Nat. Phys..

[5] Pan A (2014). Contrast enhancement in x-ray phase contrast tomography. Opt. Express.

[6] Holler M (2017). High-resolution non-destructive three-dimensional imaging of integrated circuits. Nature.

[7] Goodman JW, Lawrence RW (1967). Digital image formation from electronically detected holograms. Appl. Phys. Lett..

[8] Creath K (1985). Phase-shifting speckle interferometry. Appl. Opt..

[9] Gerchberg RW, Saxton WO (1972). A practical algorithm for the determination of phase from image and diffraction plane pictures. Optik.

[10] Fienup JR (1978). Reconstruction of an object from the modulus of its Fourier transform. Opt. Lett..

[11] Zheng GA, Horstmeyer R, Yang C (2013). Wide-field, high-resolution Fourier ptychographic microscopy. Nat. Photonics.

[12] Tian L (2014). Multiplexed coded illumination for Fourier Ptychography with an LED array microscope. Biomed. Opt. Express.

[13] Teague MR (1983). Deterministic phase retrieval: a Green’s function solution. J. Optical Soc. Am..

[14] Streibl N (1984). Phase imaging by the transport equation of intensity. Opt. Commun..

[15] Fienup JR (1982). Phase retrieval algorithms: a comparison. Appl. Opt..

[16] Bauschke HH, Combettes PL, Luke DR (2002). Phase retrieval, error reduction algorithm, and fienup variants: a view from convex optimization. J. Optical Soc. Am. A.

[17] Gerchberg RW (1986). The lock problem in the Gerchberg-, Saxton algorithm for phase retrieval. Optik.

[18] Fienup JR, Wackerman CC (1986). Phase-retrieval stagnation problems and solutions. J. Optical Soc. Am. A.

[19] Gregor, K. & LeCun, Y. Learning fast approximations of sparse coding. In *Proceedings of the 27th International Conference on Machine Learning*. (Omnipress, Haifa, 2010).

[20] Rubinstein R, Bruckstein AM, Elad M (2010). Dictionaries for sparse representation modeling. Proc. IEEE.

[21] Bao CL (2016). Dictionary learning for sparse coding: algorithms and convergence analysis. IEEE Trans. Pattern Anal. Mach. Intell..

[22] Mardani, M. et al. Recurrent generative adversarial networks for proximal learning and automated compressive image recovery. Preprint at arXiv.org/abs/1711.10046 (2017).

[23] Yang, C. Y., Ma, C. & Yang, M. H. Single-image super-resolution: a benchmark. In *Proceedings of the 13th European Conference on Computer Vision*. (Springer, Zurich, 2014).

[24] Dong C (2016). Image super-resolution using deep convolutional networks. IEEE Trans. Pattern Anal. Mach. Intell..

[25] Jin KH (2017). Deep convolutional neural network for inverse problems in imaging. IEEE Trans. Image Process..

[26] McCann MT, Jin KH, Unser M (2017). Convolutional neural networks for inverse problems in imaging: a review. IEEE Signal Process. Mag..

[27] Sinha A (2017). Lensless computational imaging through deep learning. Optica.

[28] Li S, Barbastathis G (2018). Spectral pre-modulation of training examples enhances the spatial resolution of the phase extraction neural network (PhENN). Opt. Express.

[29] Goy A (2018). Low photon count phase retrieval using deep learning. Phys. Rev. Lett..

[30] Rivenson Y (2018). Phase recovery and holographic image reconstruction using deep learning in neural networks. Light Sci. Appl..

[31] Wu YC (2018). Extended depth-of-field in holographic imaging using deep-learning-based autofocusing and phase recovery. Optica.

[32] Nguyen T (2018). Deep learning approach for Fourier ptychography microscopy. Opt. Express.

[33] Xue Y (2019). Reliable deep-learning-based phase imaging with uncertainty quantification. Optica.

[34] Kamilov US (2015). Learning approach to optical tomography. Optica.

[35] Goy A (2019). High-resolution limited-angle phase tomography of dense layered objects using deep neural networks. Proc. Natl Acad. Sci. USA.

[36] Jo Y (2019). Quantitative phase imaging and artificial intelligence: a review. IEEE J. Sel. Top. Quantum Electron..

[37] Barbastathis G, Ozcan A, Situ G (2019). On the use of deep learning for computational imaging. Optica.

[38] Daubechies I (1988). Orthonormal bases of compactly supported wavelets. Commun. Pure Appl. Math..

[39] Daubechies I (1992). Ten Lectures on Wavelets..

[40] Coifman, R. R. & Donoho, D. L. Translation-invariant de-noising. In *Wavelets and Statistics*, Vol. 103 (eds. Antoniadis, A. & Oppenheim, G.) (Springer-Verlag, New York, 1995), 120–150.

[41] Strang, G. & Nguyen, T. *Wavelets and Filter Banks*. 2nd edn. (Wellesley-Cambridge Press, Wellesley, 1996).

[42] Chan RH (2003). Wavelet algorithms for high-resolution image reconstruction. SIAM J. Sci. Comput..

[43] Daubechies I, Defrise M, De Mol C (2004). An iterative thresholding algorithm for linear inverse problems with a sparsity constraint. Commun. Pure Appl. Math..

[44] Figueiredo MAT, Nowak RD (2003). An EM algorithm for wavelet-based image restoration. IEEE Trans. Image Process..

[45] Mallat, S. *A Wavelet Tour of Signal Processing: The Sparse Way*. 3rd edn. (Amsterdam: Academic Press, 2008).

[46] Lim D, Chu KK, Mertz J (2008). Wide-field fluorescence sectioning with hybrid speckle and uniform-illumination microscopy. Opt. Lett..

[47] Mertz J (2011). Optical sectioning microscopy with planar or structured illumination. Nat. Methods.

[48] Bhattacharya D (2012). Three dimensional HiLo-based structured illumination for a digital scanned laser sheet microscopy (DSLM) in thick tissue imaging. Opt. Express.

[49] Zhu YH (2014). Low-noise phase imaging by hybrid uniform and structured illumination transport of intensity equation. Opt. Express.

[50] Han Y, Ye JC (2018). Framing U-net via deep convolutional framelets: application to sparse-view CT. IEEE Trans. Med. Imaging.

[51] Ye JC, Han Y, Cha E (2018). Deep convolutional framelets: a general deep learning framework for inverse problems. SIAM J. Imaging Sci..

[52] Pan, J. S. et al. Learning dual convolutional neural networks for low-level vision. In *Proceedings of 2018 IEEE/CVF Conference on Computer Vision and Pattern Recognition*. (IEEE, Salt Lake City, 2018).

[53] Romano Y, Elad M, Milanfar P (2017). The little engine that could: regularization by denoising (RED). SIAM J. Imaging Sci..

[54] Tikhonov AN (1963). On the solution of ill-posed problems and the method of regularization. Dokl. Akademii Nauk SSSR.

[55] Candès EJ, Tao T (2005). Decoding by linear programming. IEEE Trans. Inf. Theory.

[56] Candès EJ, Romberg J, Tao T (2006). Robust uncertainty principles: exact signal reconstruction from highly incomplete frequency information. IEEE Trans. Inf. Theory.

[57] Donoho DL (2006). Compressed sensing. IEEE Trans. Inf. Theory.

[58] Candès EJ, Romberg JK, Tao T (2006). Stable signal recovery from incomplete and inaccurate measurements. Commun. Pure Appl. Math..

[59] Eldar YC, Kutyniok G (2012). Compressed Sensing: Theory and Applications..

[60] Elad M, Aharon M (2006). Image denoising via sparse and redundant representations over learned dictionaries. IEEE Trans. Image Process..

[61] Aharon M, Elad M, Bruckstein A (2006). K-SVD: an algorithm for designing overcomplete dictionaries for sparse representation. IEEE Trans. Signal Process..

[62] Olshausen BA, Field DJ (1996). Emergence of simple-cell receptive field properties by learning a sparse code for natural images. Nature.

[63] Olshausen BA, Field DJ (1996). Natural image statistics and efficient coding. Netw. Comput. Neural Syst..

[64] Van Der Schaaf A, Van Hateren JH (1996). Modelling the power spectra of natural images: statistics and information. Vis. Res..

[65] Lewicki MS, Olshausen BA (1999). Probabilistic framework for the adaptation and comparison of image codes. J. Optical Soc. Am. A.

[66] Lewicki MS, Sejnowski TJ (2000). Learning overcomplete representations. Neural Comput..

[67] Russakovsky O (2015). ImageNet large scale visual recognition challenge. Int. J. Computer Vis..

[68] Gupta, P. et al. A modified PSNR metric based on hvs for quality assessment of color images. In *Proceedings of 2011 International Conference on Communication and Industrial Application*. (IEEE, Kolkata, 2011).

[69] Wang, Z. et al. Multiscale structural similarity for image quality assessment. In *Proceedings of the Thirty-Seventh Asilomar Conference on Signals, Systems & Computers*. (IEEE, Pacific Grove, 2003).

[70] Wang Z (2004). Image quality assessment: from error visibility to structural similarity. IEEE Trans. Image Process..

[71] Pearson K (1896). Contributions to the mathematical theory of evolution. note on reproductive selection. Proc. R. Soc. Lond..

[72] Lee Rodgers J, Nicewander WA (1988). Thirteen ways to look at the correlation coefficient. Am. Statistician.

[73] Li S (2018). Imaging through glass diffusers using densely connected convolutional networks. Optica.

[74] Deng, M., Li, S. & Barbastathis, G. Learning to synthesize: splitting and recombining low and high spatial frequencies for image recovery. Preprint at arXiv.org/abs/1811.07945 (2018).

[75] Ronneberger, O., Fischer, P. & Brox, T. U-Net: convolutional networks for biomedical image segmentation. In *Proceedings of the 18th International Conference on Medical Image Computing and Computer-Assisted Intervention*. (Springer, Munich, 2015).

[76] Bottou, L. Large-scale machine learning with stochastic gradient descent. In *Proceedings of the 19th International Conference on Computational Statistics*. (Springer, Paris, 2010).

[77] Kingma, D. P. & Ba, J. Adam: A method for stochastic optimization. In *Proceedings of the 3rd International Conference on Learning Representations*. (Conference Track Proceedings, San Diego, 2015).

[78] Hinton, G. E. Learning translation invariant recognition in a massively parallel networks. In *Proceedings of the International Conference on Parallel Architectures and Languages Europe*. (Springer-Verlag, Eindhoven, 1987).

[79] Johnson, J., Alahi, A. & Li, F. F. Perceptual losses for real-time style transfer and super-resolution. In *Proceedings of the 14th European Conference on Computer Vision*. (Springer, Amsterdam, 2016).

[80] Ledig, C. et al. Photo-realistic single image super-resolution using a generative adversarial network. In *Proceedings of the IEEE Conference on Computer Vision and Pattern Recognition*. (IEEE, Honolulu, 2017).

[81] Li, S., Barbastathis, G. & Goy, A. Analysis of phase extraction neural network (PhENN) performance for lensless quantitative phase imaging. *Proc. SPIE 10887, Quantitative Phase Imaging V, 108870T* (4 March 2019).

[82] Goy, A. et al. The importance of physical pre-processors for quantitative phase retrieval under extremely low photon counts. *Proc. SPIE 10887, Quantitative Phase Imaging V, 108870S* (4 March 2019).

