# Supplementary material for: Learning to synthesize: robust phase retrieval at low photon counts
Source: Light Sci Appl. 2020 Mar 9;9:36. doi: 10.1038/s41377-020-0267-2 (PMC7062747; doi:10.1038/s41377-020-0267-2)
Supplement: Supplementary file 1 — Supplementary Information [file 41377_2020_267_MOESM1_ESM.pdf]

Learning to Synthesize: Robust Phase Retrieval at Low Photon Counts: Supplementary Material

Mo Deng[1,*], Shuai Li[2], Alexandre Goy[3], Iksung Kang[1], and George Barbastathis[4,5]

[1]Department of Electrical Engineering and Computer Science, Massachusetts Institute of Technology, Cambridge, MA 02139, USA
[2]Sensebrain Technology Limited LLC, 2550 N 1st Street, Suite 300, San Jose, CA 95131, USA
[3]Omnisens SA, Riond Bosson 3, 1110 Morges, VD, Switzerland
[4]Department of Mechanical Engineering, Massachusetts Institute of Technology, Cambridge, MA 02139, USA
[5]Singapore-MIT Alliance for Research and Technology (SMART) Centre, Singapore 117543, Singapore
[*]Corresponding author: modeng@mit.edu

February 12, 2020

1 Deep neural network structures and training details

The architecture of DNN-L (and DNN-H) is shown in Fig S1. It assumes an *encoder-decoder* structure where the *encoder* consists 4 Down-Residual blocks (DRBs) to gradually reduce the size (length and width) of the feature maps to extract compressed representations of the signal to preserve high-level (or equivalently, low spatial frequency) features; subsequently, the *decoder* component, comprises of 4 Up-Residual blocks (URBs) and two (constant-size) Residual blocks (RBs) to expand the size of the feature maps to form the final reconstruction. Critically important for high spatial frequency preservation, skip connections are used to bypass the feature maps from the *encoder* to the corresponding layers of the same size in the *decoder*. This particular DNN architecture, also referred to as the Residual U-net [1], is used in [2] and has been proven to be versatile to many different image-related applications.

Fig S2 shows the general architecture of DNN-S. DNN-S trains to take in two preliminary reconstructions $\hat{f}^{\mathrm{LF}}$ and $\hat{f}^{\mathrm{HF}}$, where $\hat{f}^{\mathrm{HF}}$ is bypassed to the near final stage of DNN-S by residual (additive) skip connections and $\hat{f}^{\mathrm{LF}}$ is mapped by an *encoder-decoder* architecture to a variant of itself to compensate the high-frequency artifacts and low-frequency distortions in $\hat{f}^{\mathrm{HF}}$ to produce the synthesized final reconstruction $\hat{f}$.

Fig S3 shows more details of Up-residual blocks (URBs), Down-residual blocks (DRBs) and Residual blocks (RBs). All convolutional and convolutional transpose kernels are 3×3, except for 2×2 kernels in the side-branch Convolutional Transpose in Residual upsampling units and the 1×1 (1D convolution) kernels in the side-branch of Residual units.

The simulation is conducted on a Nvidia GTX1080 GPU using the open source machine learning Platform TensorFlow. Each of DNN-L, DNN-H and DNN-S is trained for 20 epochs on 9500 training examples. The Adam optimizer [3], with learning rate being 0.001 and exponential decay rate for the first and second moment estimates being 0.9 and 0.999 (β_1=0.9, β_2=0.999). The batch size is 5. The training of each DNN takes about 2 hours. Noting that DNN-L and DNN-H can be trained completely in parallel, the total training time is around 4 hours.

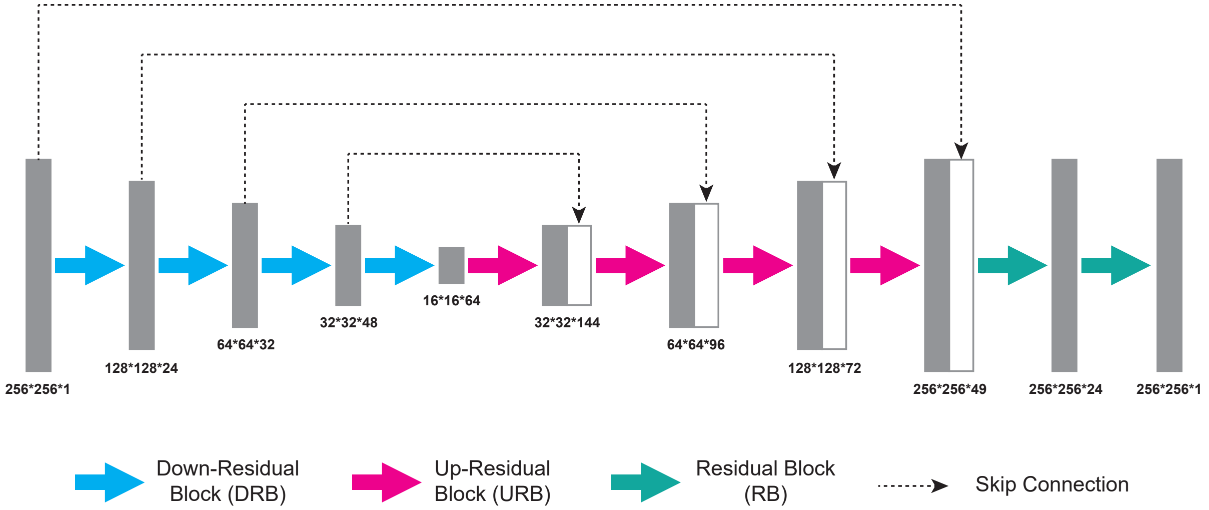

Figure S1: General architecture of DNN-L (and DNN-H)

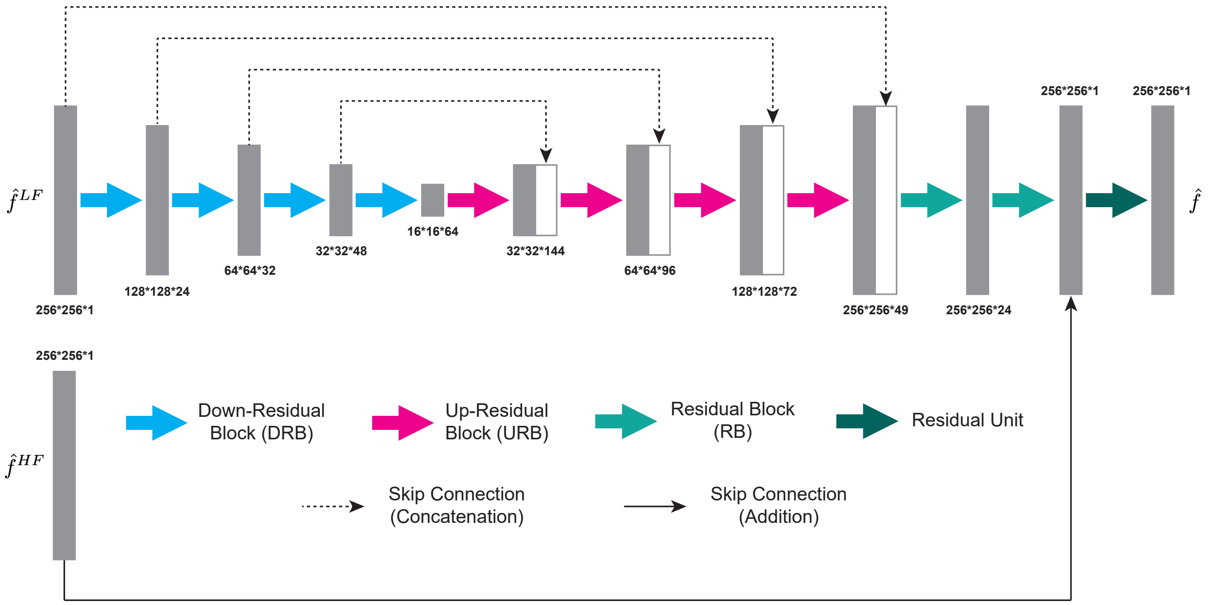

Figure S2: General structure of DNN-S

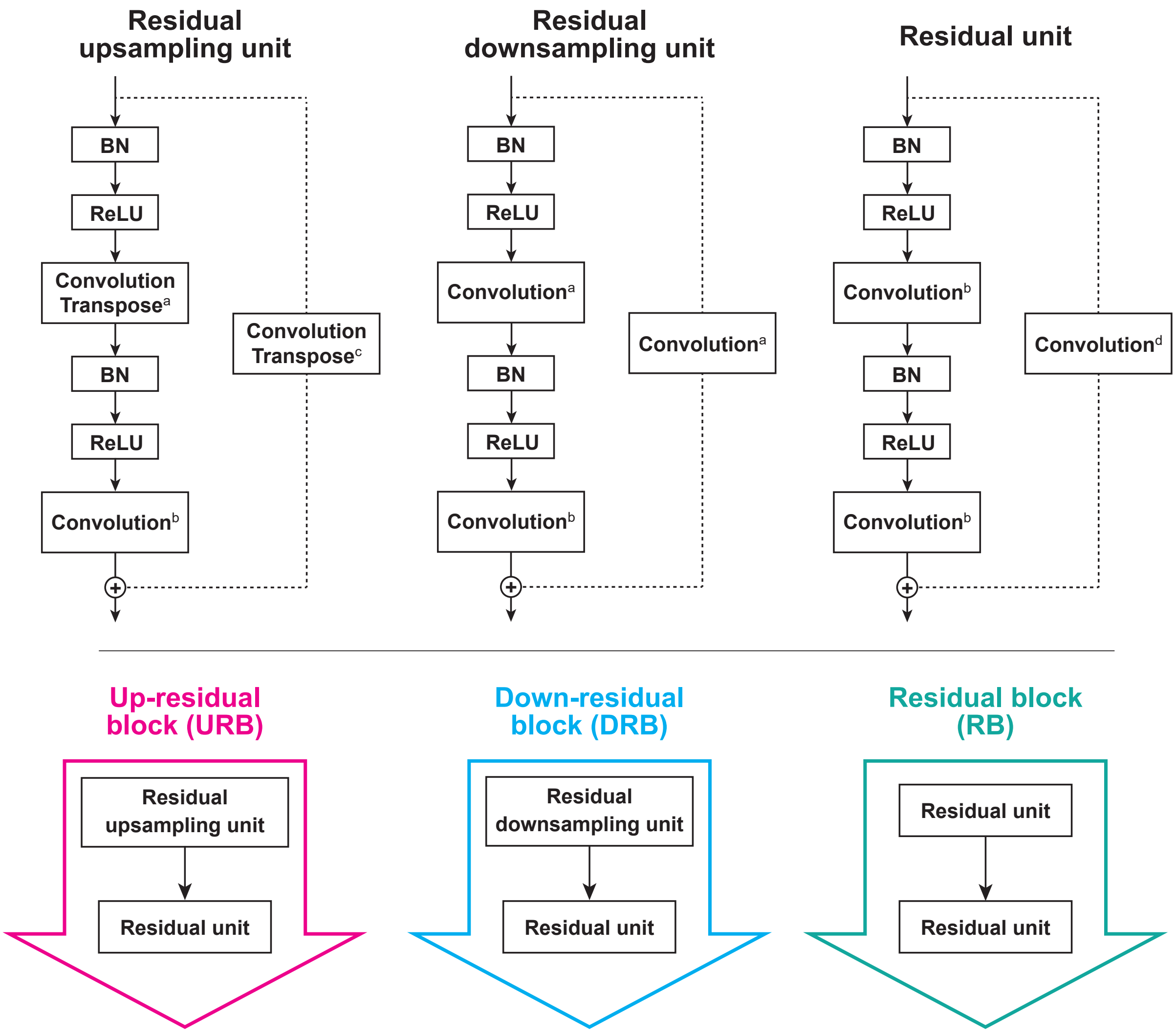

Figure S3: Detailed Structures of DRBs, URBs, RBs. Superscripts a - d denote different kernel size and strides, listed as follows: a) Kernel size: (3, 3), strides: (2, 2). b) Kernel size: (3, 3), strides: (1, 1). c) Kernel size: (2, 2), strides: (2, 2). d) Kernel size: (1, 1), strides: (1, 1).

2 More reconstruction examples

In Fig. S4, we show more examples of comparison between the approximant $\hat{f}^*$,reconstructions $\hat{f}^{\mathrm{LF}}$, $\hat{f}^{\mathrm{HF}}$, $\hat{f}$ and the ground truth f, for $p = 1$ photon/pixel and $q = 0.5$. Similar to the examples shown in the main manuscript, we see that the DNN-L output, $\hat{f}^{\mathrm{LF}}$ is reliable in low spatial frequency band; whereas DNN-H output, $\hat{f}^{\mathrm{HF}}$ is reliable in the high spatial frequency band, and the final reconstruction, $\hat{f}$ has better overall quality than both $\hat{f}^{\mathrm{LF}}$ and $\hat{f}^{\mathrm{HF}}$.

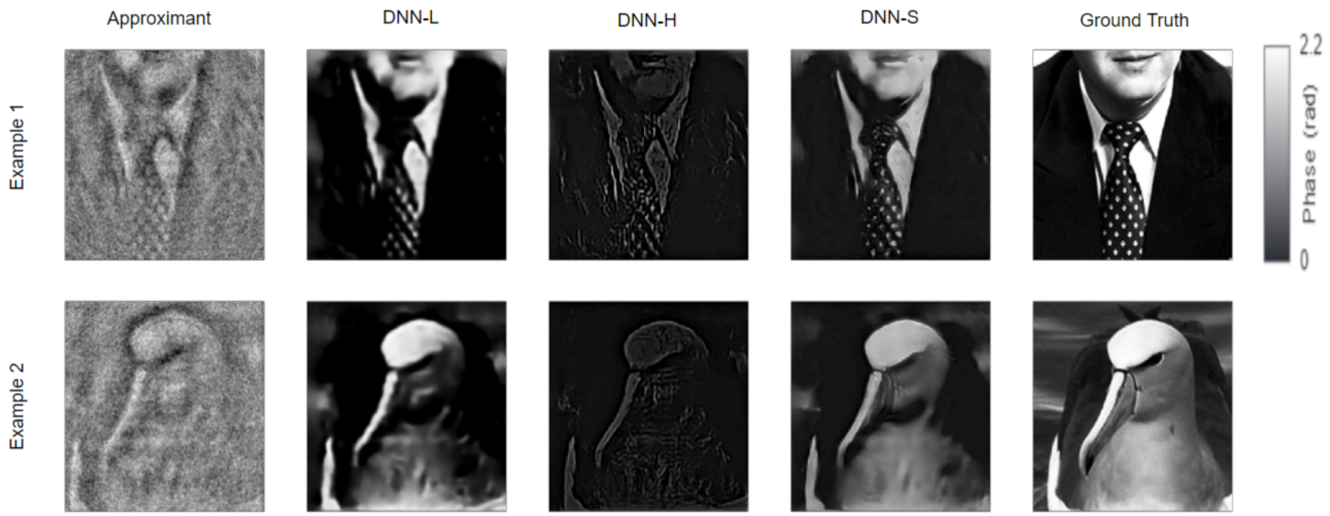

Figure S4: More examples of components of LS-DNN, 1 photon/pixel

3 1D cross-section of PSDs for 10 photon/pixel

Figure. S5 shows the average (normalized) 1D (diagonal) cross-section of the approximant $\hat{f}^*$, DNN-L output $\hat{f}^{\mathrm{LF}}$, DNN-H output $\hat{f}^{\mathrm{HF}}$, final output $\hat{f}$ and ground truth f, for the case of 10 photons/pixel. Similar to the 1 photon/ pixel case, we see $\hat{f}^{\mathrm{LF}}$ and $\hat{f}^{\mathrm{HF}}$ are more reliable in low and high frequency bands, respectively and the synthesized output, $\hat{f}$ matches the ground truth better than each preliminary reconstruction on the entire spectrum.

4 More quantitative performance comparisons

We show in Figs. S6, S7, S8, quantitative performances (according to PSNR, SSIM and PCC, respectively) of the approximant $\hat{f}^*$ (input to the LS-DNN), DNN-L output, $\hat{f}^{\mathrm{LF}}$, and the final reconstruction $\hat{f}$, for each of the 500 test images randomly chosen from the test set (a total of 500 images), with both $p = 1$ and $p = 10$ and under $q = 0.5$. The comparisons show, except for very few anomalies, $\hat{f}$ outperforms $\hat{f}^{\mathrm{LF}}$ (and $\hat{f}^*$) for every image, and according to all three metrics, for both 1 photon/ pixel and 10 photons/pixel. Therefore, improvements over [2] (performance of DNN-L) have been corroborated.

5 Qualitative comparison with DNN-L-3

We demonstrate that the improvement of our reconstruction $\hat{f}$ over [2] ($\hat{f}^{\mathrm{LF}}$ in this paper) should not be attributed to increased computational capacity by comparing $\hat{f}$ with the reconstruction

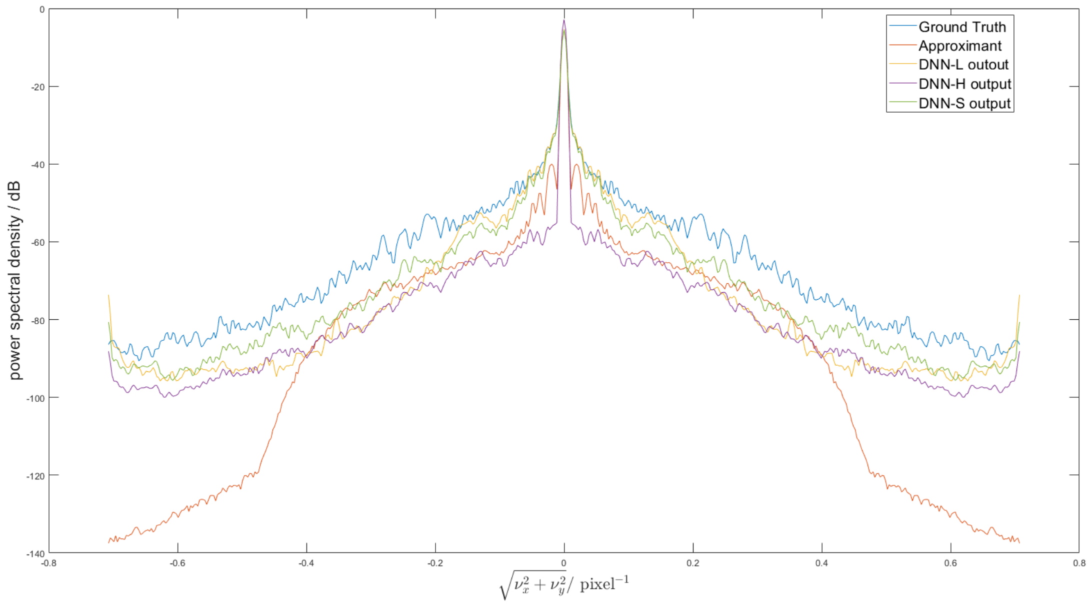

Figure S5: 1D cross-section of average PSD, 10 photons/pixel

obtained with a separate DNN-L-3, which assumes the similar Residual U-Net [1] structure, but has twice as many feature maps in each convolutional layer as its DNN-L counterpart (except for those in Residual Blocks), which approximately equalizes the total number of trainable parameters in the LS-DNN architecture and in DNN-L-3. Unlike LS-DNN, DNN-L-3, is trained with the un-filtered examples and with NPCC as the loss function. Quantitative comparison of LS-DNN and DNN-L-3 has been given in the main manuscript.

In Fig. S9, we see at both 1 photon/pixel and 10 photons/pixel, DNN-L-3, could not achieve performances comparable to LS-DNN (reconstructions obtained under $q = 0.5$ is shown as the performance of LS-DNN).

6 Resolution test for LS-DNN

In this section, we investigate the spatial resolution (according to the Rayleigh criterion) of various reconstructions in LS-DNN and investigate the role of parameter q in the resolution.

The objects used are dot patterns with spacing of D pixels between each pair of dots. In this paper, we test D in the range from 2 to 8 pixels. We obtain the intensity measurement of each of these dot patterns and find the minimum D, such that the dots are resolvable (according to the Rayleigh criterion) in the reconstruction and define such D as the resolution. For example, in Fig. S10, we see that for $q = 0.5$ and spacing $D = 4$, the neighbouring dots are resolvable in DNN-H and DNN-S output, but not in the DNN-L output. Simulations (not shown here) also indicate dots with $D \leq 3$ are not resolvable by DNN-H or DNN-S, so DNN-H and DNN-S both resolve 4 pixels. This improves over DNN-L, or [4], which achieved the resolution of $D = 6$ pixels (resolution characterization of [4] was conducted in [5]).

For completeness, we include in Table S1 resolutions achieved by DNN-L, DNN-H and DNN-S, for $q = 0.4, 0.5, 0.6$, respectively. As expected, LS-DNN method (DNN-S) achieves better resolution than DNN-L, trained with un-filtered examples. Although in all q, DNN-S does not achieve

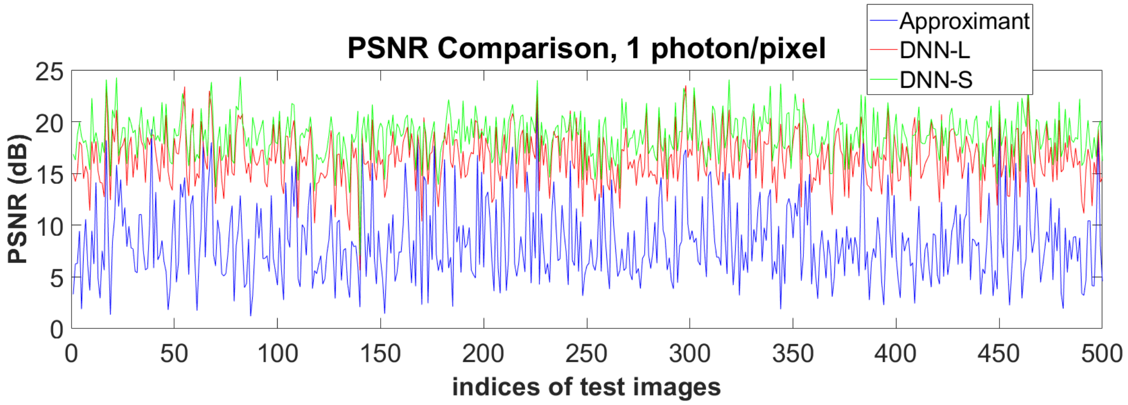

(a) PSNR comparison, 1 photon/pixel

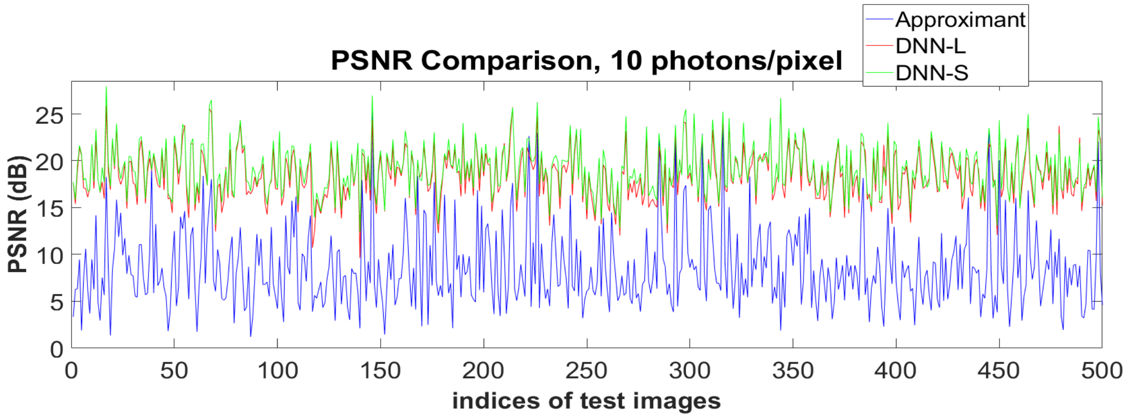

(b) PSNR comparison, 10 photons/pixel

Figure S6: PSNR comparison ($q = 0.5$)

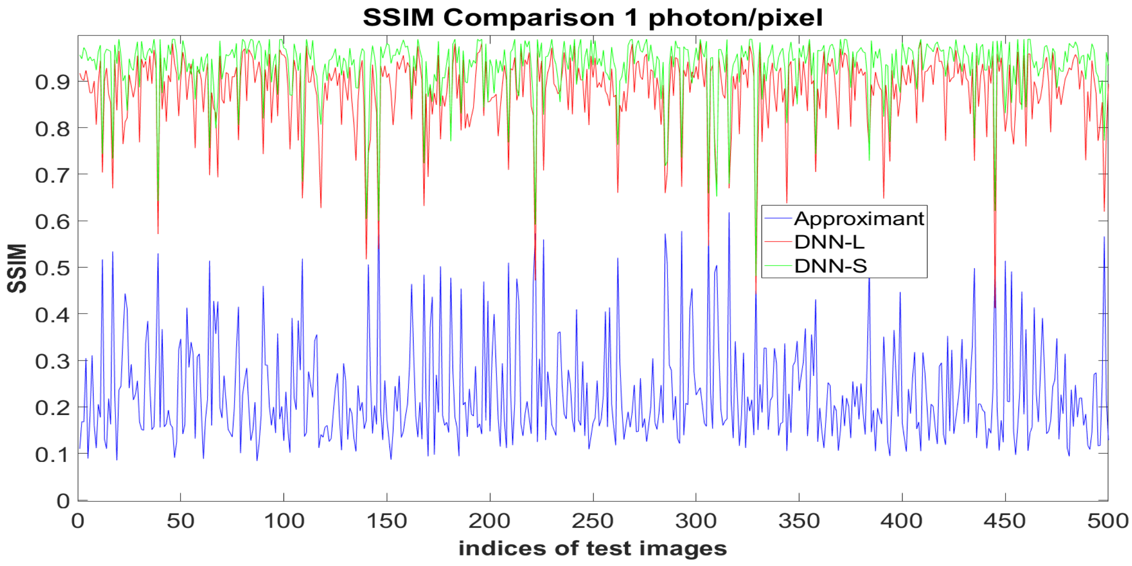

(a) SSIM comparison, 1 photon/pixel

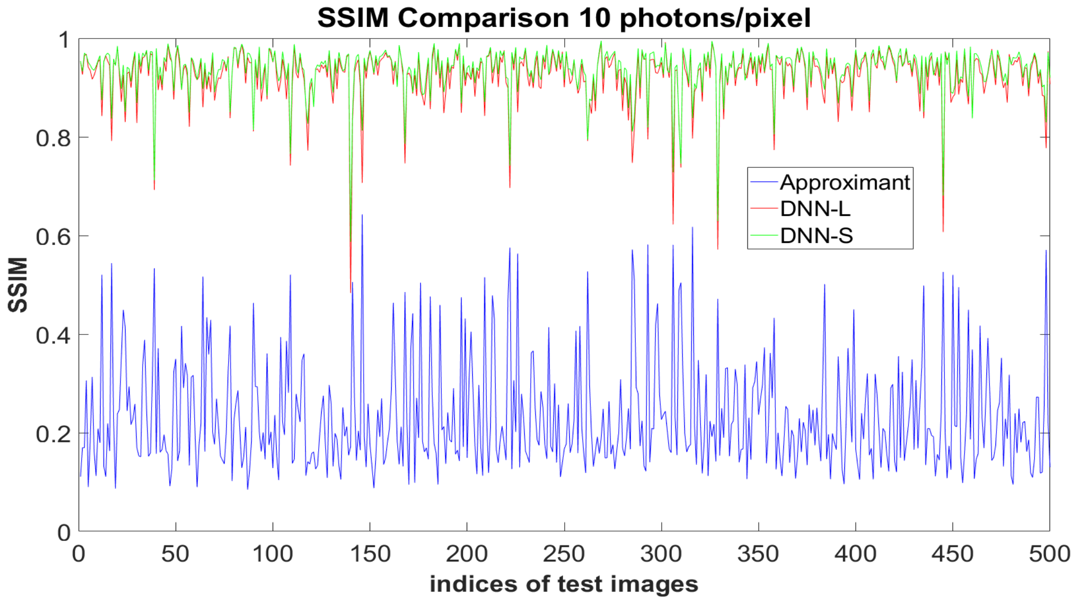

(b) SSIM comparison, 10 photons/pixel

Figure S7: SSIM comparison ($q = 0.5$)

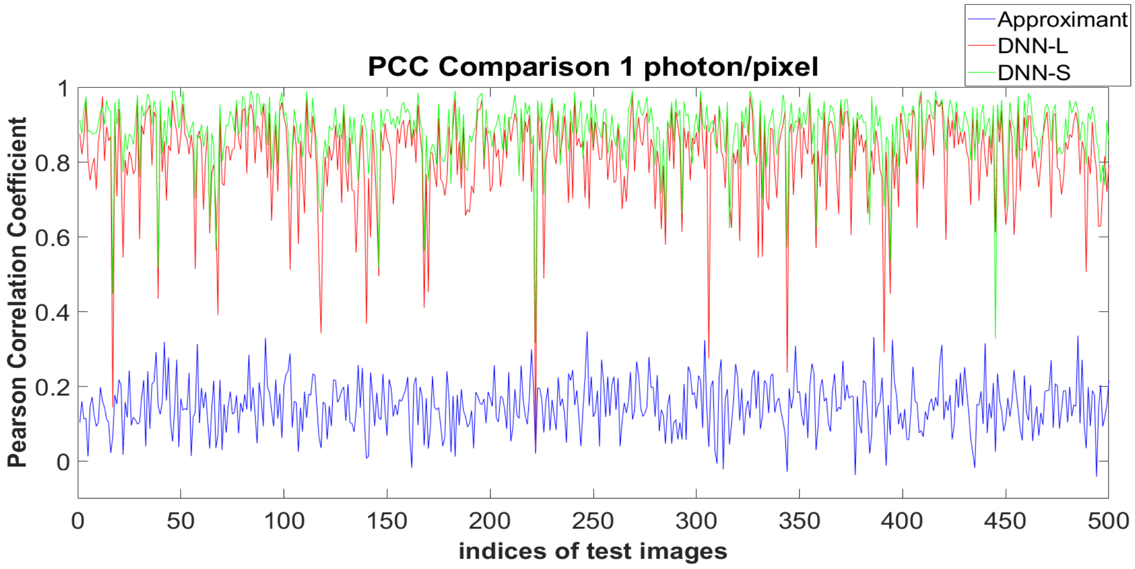

(a) PCC comparison, 1 photon/pixel

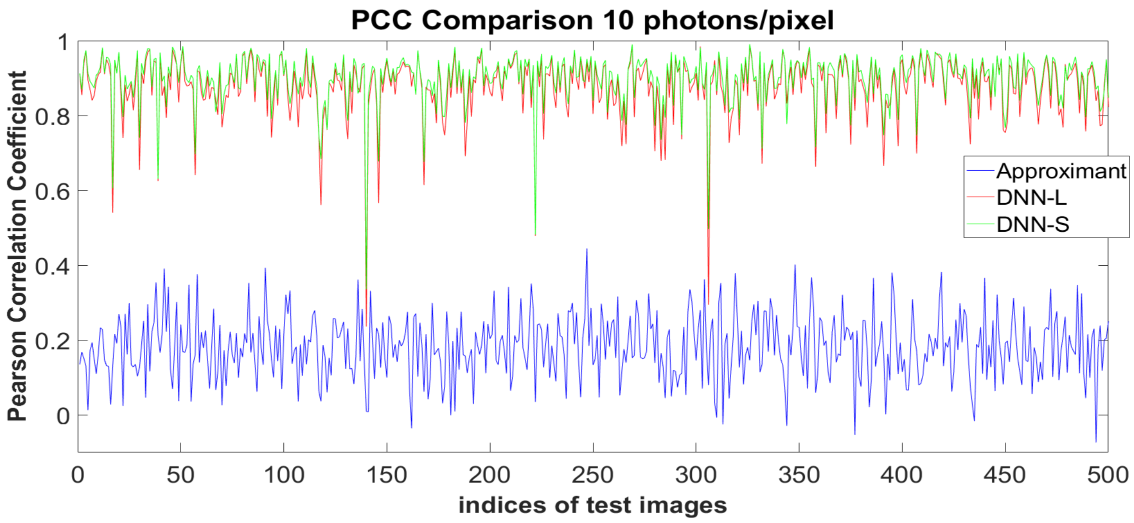

(b) PCC comparison, 10 photons/pixel

Figure S8: PCC comparison ($q = 0.5$)

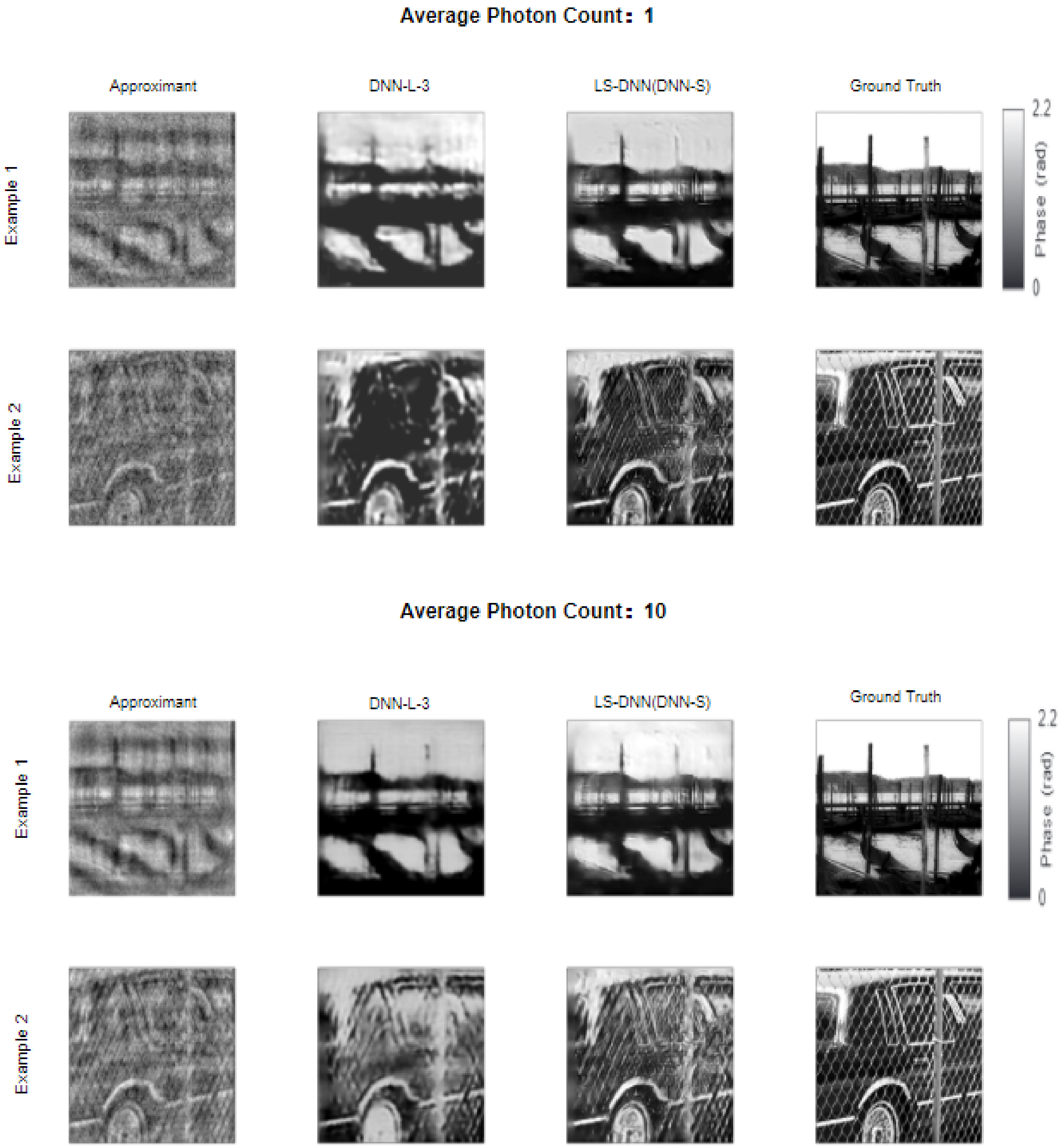

Figure S9: Comparison of DNN-L-3 reconstruction with LS-DNN final reconstruction $\hat{f}$ ($q = 0.5$)

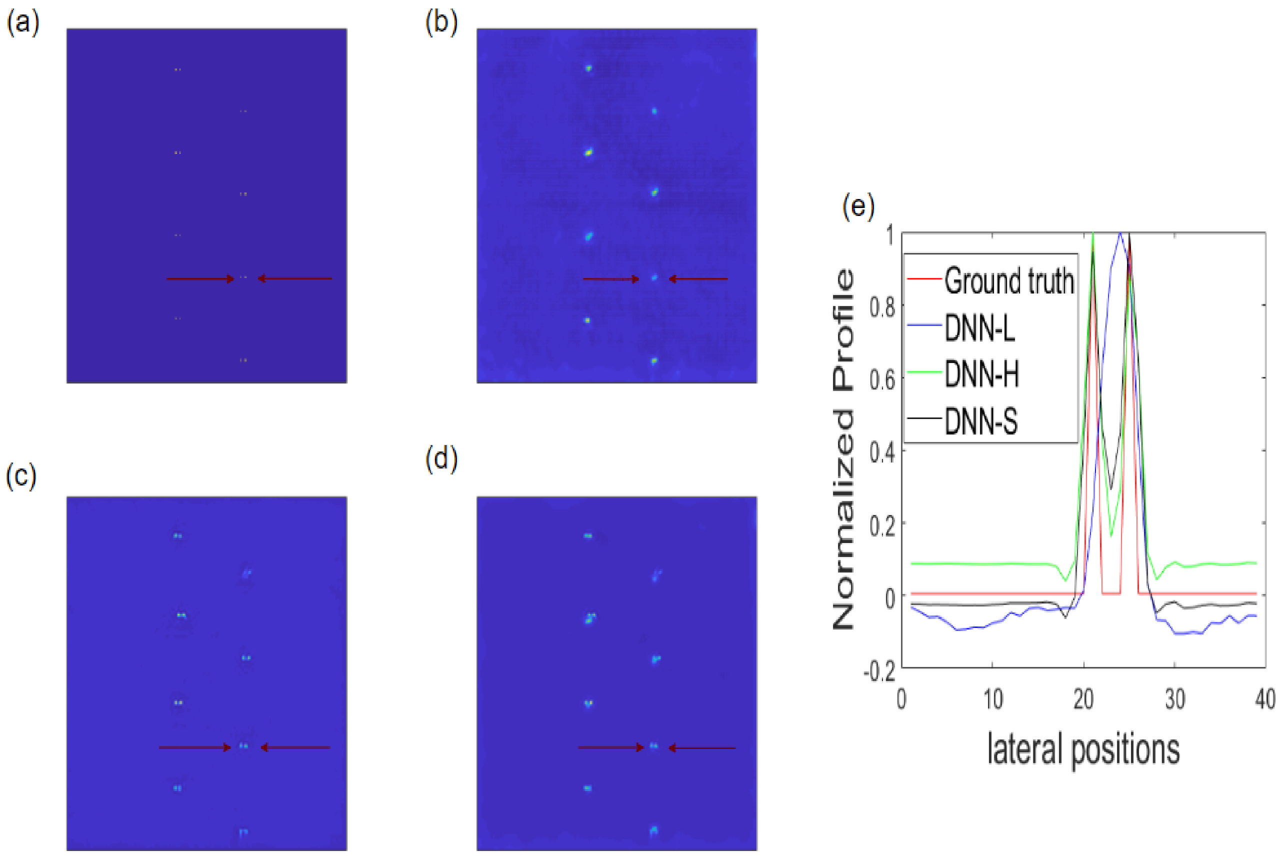

Figure S10: Resolution test for DNN-L, DNN-H and DNN-S outputs ($q = 0.5$). (a). dot pattern with spacing $D = 4$cm (ground truth) (b). DNN-L reconstruction. (c) DNN-H reconstruction (d) DNN-S reconstruction. (e).1D cross-section of the neighbourhood indicated by the red arrows in (a)-(d).

further improvement in resolution over DNN-H, it is absolutely necessary as it helps improve the overall fidelity [6], as is also shown by the noisy cases in Figures 2, 3, 4, 5, 6, and Table 1 in the main manuscript. Ultimately, it is the fidelity of reconstructions, rather than the resolution, that matters the most in the application of quantitative phase retrieval.

DNN-L: 6 pixels				
	$q = 0.4$	$q = 0.5$	$q = 0.6$	$q = 0.7$
DNN-H	4 pixels	4 pixels	3 pixels	3 pixels
DNN-S	4 pixels	4 pixels	3 pixels	3 pixels

Table S1: Resolution comparison for various reconstructions in LS-DNN.

7 Additional details about the Fresnel propagation operator

In this section, we provide more details about the implementation of the optical apparatus and its numerical simulation with digital Fresnel transforms. Please refer to Fig. 7 in the main manuscript

for the description of the optical apparatus.

The optical apparatus includes a telescope that scales down the size of the object by a factor of $2.3\times$. This was done to reduce the spatial extent of the image and enhance the diffraction effects so that shorter distances would be required to achieve comparable Fresnel numbers. With this particular reduction, the raw image (256 by 256 pixel array on the SLM) approximately fits the size of the camera detector. The raw data from the detector is a 1004×1002 array that was first extended to 1024×1024 by repeating the edge values into the extended area. The image is then reduced to a 256×256 array by 4×4 pixel summation pooling. Thus, the space-bandwidth product (SBP) of the effective measurement g matched the SBP of the object f. However, this creates another difficulty: *i.e.* the fact that the pixel size in the detector plane in general does not match the pixel size obtained from a digital Fresnel propagation from the object plane. Indeed, recall that, when performing Fresnel propagation with the FFT operator, using the following formula:

$$\mathbf{F}_z[\psi] = \frac{e^{ikz}}{i\lambda z} e^{\frac{ik}{2z}(x'^2+y'^2)} \mathrm{FFT}\left\{\psi(x,y) e^{\frac{ik}{2z}(x^2+y^2)}\right\} \Delta x \Delta y, \tag{1}$$

the pixel size in the object plane (Δx) and detector plane ($\Delta x'$) obey the following the relationship:

$$\lambda z = (\mathrm{SBP})\, \Delta x \Delta x'. \tag{2}$$

In our case, these quantities are fixed as $\Delta x = 15.7\ \mu$m (*i.e.* the SLM pixel size, 36 μm, reduced by $2.3\times$), $\Delta x' = 8\ \mu$m, (SBP) = 256 and $z = 400$ mm, and do not satisfy (2). We circumvent this difficulty by introducing an intermediate plane, which we call a virtual plane hereafter, and performing two Fresnel propagation operations: one from the object plane to the virtual plane (over a distance z_{v1}), and a second one from the virtual plane to the detector plane (over a distance z_{v2}). The position along the z axis of the virtual plane, which we call virtual distance z_{v}, offers an additional degree of freedom that we can use to satisfy equation (2). The algebraic sum of z_{v1} and z_{v2} should equal z. If we denote by $\Delta x''$ the pixel size in the virtual plane, we have:

$$\lambda z_{\mathrm{v1}} = (\mathrm{SBP}) \Delta x \Delta x'' \tag{3}$$
$$\lambda z_{\mathrm{v2}} = (\mathrm{SBP}) \Delta x' \Delta x''. \tag{4}$$

z_{v1} and z_{v2} are then given by the following equations:

$$z_{\mathrm{v1}} \Delta x' = z_{\mathrm{v2}} \Delta x \tag{5}$$
$$z_{\mathrm{v1}} + z_{\mathrm{v2}} = z. \tag{6}$$

This mathematical device allows us to perform Fresnel propagation and pixel size interpolation at the same time.

8 More information on the data acquisition

The SLM is LC2012 by Holoeye. This is a transmissive device with 36 microns square pixels. The phase and amplitude response of the SLM was measured with an interferometer and is given in Fig. S11. The phase depth of 2.2 rad of the examples comes from the calibration of the transmissive SLM, where a phase shift of 2.2 rad corresponds to a level of 255 in the ground truth images.

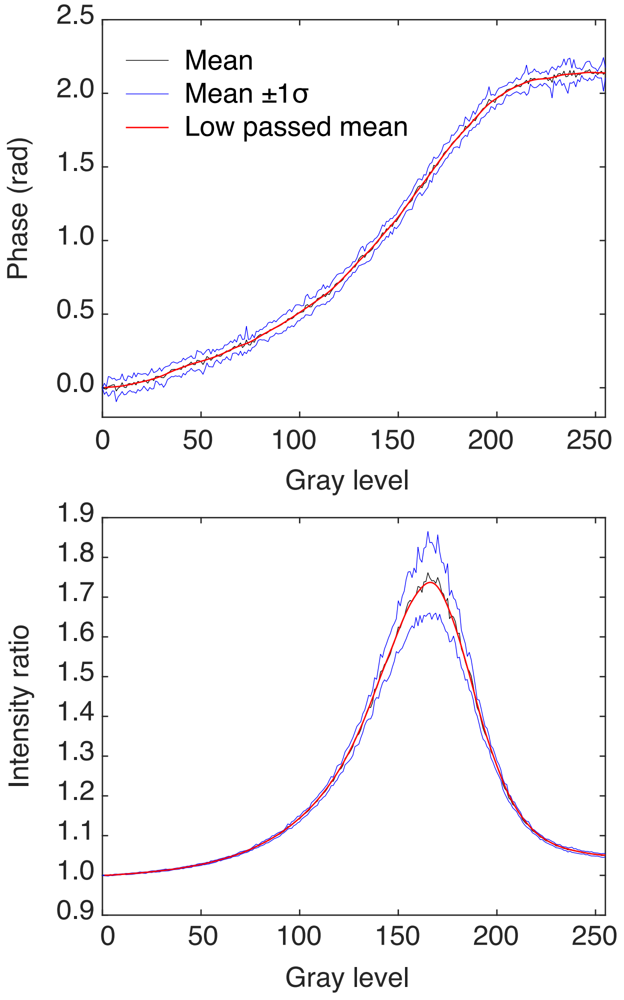

Figure S11: The phase response (top) and the amplitude response (below) of the SLM [2].

9 More on the training of various DNNs

In Fig. S12, we show the training curves for $p = 1$ and $q = 0.5$. We see that, the validation loss is, in all three cases, only slightly worse than that of the training loss when the training is stabilized, an indication of no overfitting or underfitting and therefore, good generalization ability of the LS-DNN model.

Moreover, in Table S2, we show the relationship between the validation error versus the size of the training set. As we can see, the grow of the training set size helps the most when the training set size is below 5000 examples. Therefore, if the access to sufficient training data is the primary concern, instead of enabling a fair comparison with [2], we could have used approximately half of the training data (5000 examples), to achieve performances relatively close to what we have in this paper. Also from this table, we judge that making the training set even larger unnecessary as further improvement in LS performance is likely incremental, and therefore insufficient to justify the additional training time needed.

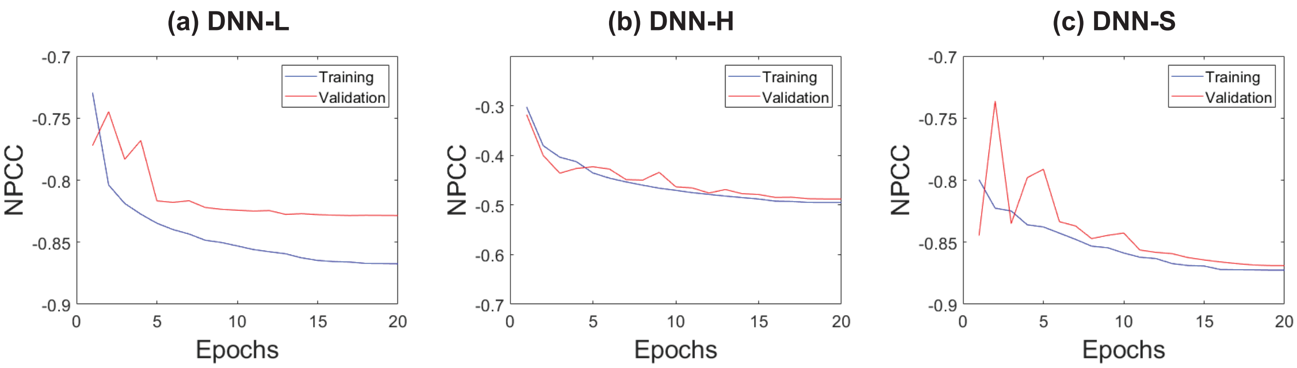

Figure S12: Training curves for (a) DNN-L (b) DNN-H and (c) DNN-S, of $p = 1$ and $q = 0.5$

Number of training examples	DNN-L	DNN-S
500	0.6927 ± 0.1375	0.6932 ± 0.1378
800	0.7181 ± 0.1198	0.7362 ± 0.1148
1000	0.7214 ± 0.1104	0.7443 ± 0.1068
2000	0.7348 ± 0.1070	0.7638 ± 0.0978
3000	0.7695 ± 0.1079	0.7926 ± 0.0924
5000	0.7911 ± 0.0884	0.8209 ± 0.0838
7500	0.8241 ± 0.0872	0.8539 ± 0.0837
9500 (in the paper)	0.8285 ± 0.0861	0.8659 ± 0.0820

Table S2: Average PCC of reconstructions of DNN-L and DNN-S, based on the validation set of 450 images. Each term takes the form of 'average PCC ± standard deviation'.

10 More quantitative metrics for evaluating the performance of LS-DNN

In this section, we investigate the performance of LS with another two quantitative metrics, *i.e.* the root mean square error (RMS) and the peak-to-valley error (PV). Let i, j be the indices of pixels for the estimated phase object $\hat{f}$ and the corresponding ground truth f, both of size $N \times N$. The RMS error can be defined as,

$$\mathrm{RMS} = \frac{\sqrt{\frac{1}{N^2}\sum_{i,j}(\hat{f}_{i,j} - f_{i,j})^2}}{2\pi}, \tag{7}$$

and the unit is wavelength. We computed the RMS according to the 500 ImageNet test images and the results shown in Table S3.

Moreover, PV error is also frequently used to assess the phase retrieval performance. In this context, we think it is most applicable to region-wise uniform objects, instead of the ImageNet images as we used in the paper. In Fig. S13, we show one example of such region-wise uniform phase objects. We created a synthetic training set consisting 5000 region-wise constant phase objects and conduct the experiment the same way as described in the manuscript (under $p = 1$ photon/pixel). During the test stage, we predict the reconstructions based on the object in Fig. S13. For each uniform (nonzero) region of the ground truth, let $a_{\max}$ and $a_{\min}$ (both in rad) be the largest and smallest value within this region in the reconstruction. Then, in wavelength, peak-to-valley (PV) error can be defined as $\frac{a_{\max}-a_{\min}}{2\pi}$ wavelength. If there are multiple regions with the same ground truth value, we report the largest regional PV error among these regions. The results, available in Table S4, show that the performance of LS is very good, which is well anticipated as the constrained

	Average RMSE ± std.dev	
	$p = 1$	$p = 10$
Approximant $\hat{f}^*$	0.1401 ± 0.0568	0.1398 ± 0.0568
DNN-L output $\hat{f}^{\mathrm{LF}}$	0.0568 ± 0.0160	0.0420 ± 0.0134
DNN-S output $\hat{f}$ ($q = 0.1$)	0.0458 ± 0.0133	0.0405 ± 0.0115
DNN-S output $\hat{f}$ ($q = 0.2$)	0.0446 ± 0.0129	0.0404 ± 0.0120
DNN-S output $\hat{f}$ ($q = 0.3$)	0.0441 ± 0.0142	0.0402 ± 0.0118
DNN-S output $\hat{f}$ ($q = 0.4$)	0.0424 ± 0.0119	0.0390 ± 0.0119
DNN-S output $\hat{f}$ ($q = 0.5$)	0.0413 ± 0.0117	0.0389 ± 0.0120
DNN-S output $\hat{f}$ ($q = 0.6$)	0.0415 ± 0.0131	0.0390 ± 0.0117
DNN-S output $\hat{f}$ ($q = 0.7$)	0.0442 ± 0.0116	0.0391 ± 0.0122
DNN-S output $\hat{f}$ ($q = 0.8$)	0.0458 ± 0.0130	0.0391 ± 0.0121
DNN-S output $\hat{f}$ ($q = 1$)	0.0482 ± 0.0121	0.0399 ± 0.0122
DNN-S output $\hat{f}$ ($q = 2$)	0.0572 ± 0.0386	0.0407 ± 0.0122

Table S3: RMSE (unit wavelength) of reconstructions of Approximant, DNN-L and DNN-S, based on a test set of 500 images. Each entry takes the form of 'average ± standard deviation'.

structure of the objects offers strong prior information which makes the LS algorithm testing on the same class of objects even more efficient.

11 Results on objects with larger phase depth

To investigate whether the performance of the proposed LS method depends on the phase depth of the objects, we selected another configuration of our transmissive SLM (LC2012 by Holoeye) that allows greater phase depth (to around 1.5π rad). Fortunately, this configuration introduces only minimal intensity modulation. The phase and intensity modulation of this configuration is shown in Fig. S14.

With this configuration, we conducted the same experiment as shown in the main manuscript on a new dataset, Face-LFW (for human faces), for $p = 1$ and $q = 0.5$. Quantitative comparison

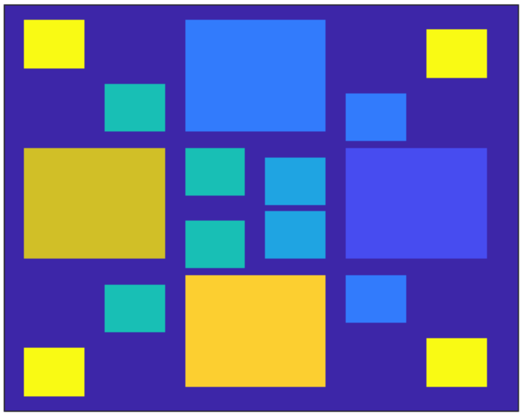

Figure S13: Synthetic phase object (ground truth) to compute PV error.

Ground Truth of the region (rad)	Peak-to-Valley Error (wavelength)
0.0307	0.0009
0.1343	0.0025
0.3243	0.0056
0.5752	0.0082
3.0014	0.0203
4.2581	0.0315
4.5932	0.0264

Table S4: Peak-to-valley error of the LS algorithm ($p = 1$ photon/pixel and $q = 0.5$).

of reconstructions of DNN-L, DNN-S and the Approximant is shown in Table S5 below. From the current results, the enlarged phase depth did not seem to have jeopardized the performance. We reserve the exploration on the full 2π rad and on other classes of objects for future investigations.

Performance of LS-DNN on phase depth 1.5π rad. ($p = 1$, $q = 0.5$).				
	PCC ± std. dev	PSNR± std.dev.	SSIM ± std.dev	RMSE±std.dev
Approximant	0.0122 ± 0.1305	10.9627 ±2.4597	0.4389 ± 0.0729	0.2190 ± 0.0701
DNN-L reconstruction	0.9263 ± 0.0279	21.3002 ±2.9396	0.9612 ± 0.0213	0.0674 ± 0.0224
DNN-S reconstruction	0.9365 ± 0.0278	21.3732 ±2.8238	0.9688 ± 0.0232	0.0672 ± 0.0219

Table S5: Quantitative results of LS on dataset with larger phase depth (1.5π rad).

12 Quantitative metrics for cross-domain generalization

In this section, we present the quantitative comparison for the cross-domain generalization discussed in Section 2 of the main manuscript (Fig.7 main manuscript). From Table S6, we clearly see that direct cross-domain generalization would certainly incur some level of degradation in the performance(compared to if the training was done on the same domain as the test examples), but such degradation is much less severe if the model was trained on the set with broader prior, *e.g.* ImageNet, and tested on a set with more constrained priors, *e.g.* MNIST, than the opposite.

PCC — Training Set / Test Set	ImageNet	MNIST
ImageNet	0.869± 0.112	0.437 ± 0.199
MNIST	0.817 ± 0.050	0.922 ± 0.062

Table S6: Quantitative metric (PCC) of cross-domain generalization of LS. Each entry takes the form of mean ± std. deviation.

References

[1] O. Ronneberger, P.Fischer, and T. Brox. U-net: Convolutional networks for biomedical image segmentation. In *Medical Image Computing and Computer-Assisted Intervention (MICCAI)*, volume 9351 of *LNCS*, pages 234–241. Springer, 2015. (available on arXiv:1505.04597 [cs.CV]).

[2] Alexandre Goy, Kwabena Arthur, Shuai Li, and George Barbastathis. Low photon count phase retrieval using deep learning. *Phys. Rev. Lett.*, 121:243902, 2018.

[3] D. P. Kingma and J. Lei Ba. Adam: A method for stochastic optimization. In *International Conference on Learning Representations (ICLR)*, 2015.

[4] A. Sinha, Justin Lee, Shuai Li, and G. Barbastathis. Lensless computational imaging through deep learning. *Optica*, 4:1117–1125, 2017.

[5] Shuai Li and G. Barbastathis. Spectral pre-modulation of training examples enhances the spatial resolution of the phase extraction neural network (PhENN). *Opt. Express*, 26(22):29340–29352, 2018.

[6] Mo Deng, Shuai Li, and George Barbastathis. Learning to synthesize: splitting and recombining low and high spatial frequencies for image recovery. *arXiv preprint arXiv:1811.07945*, 2018.

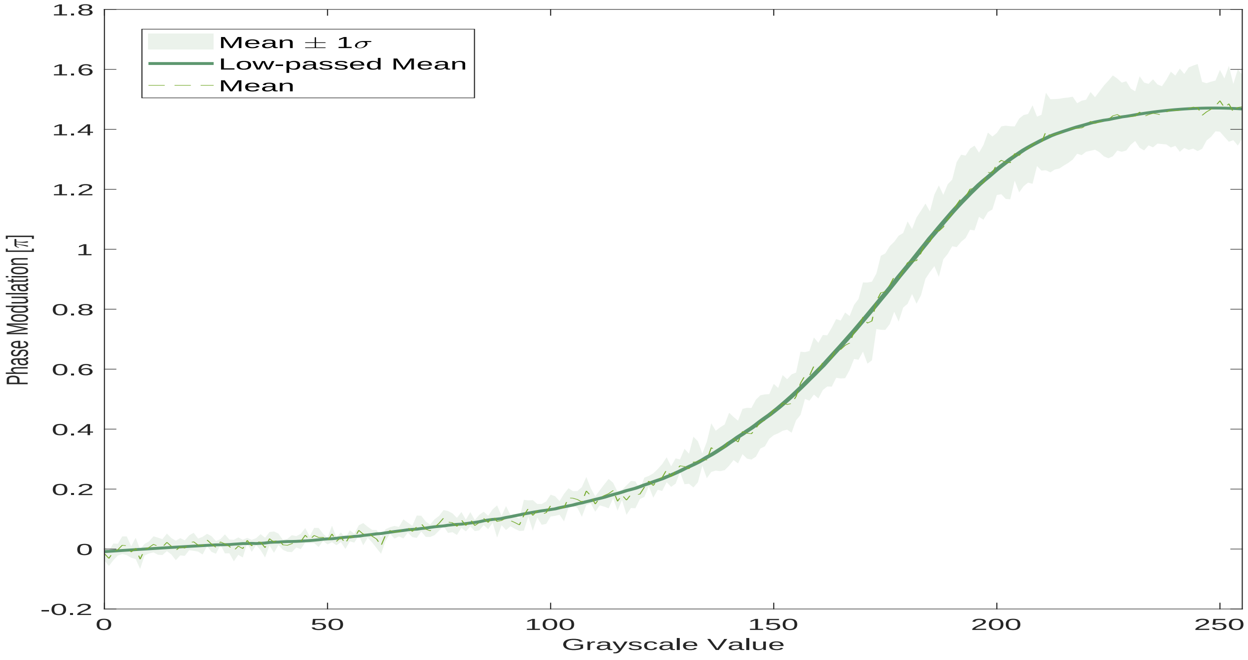

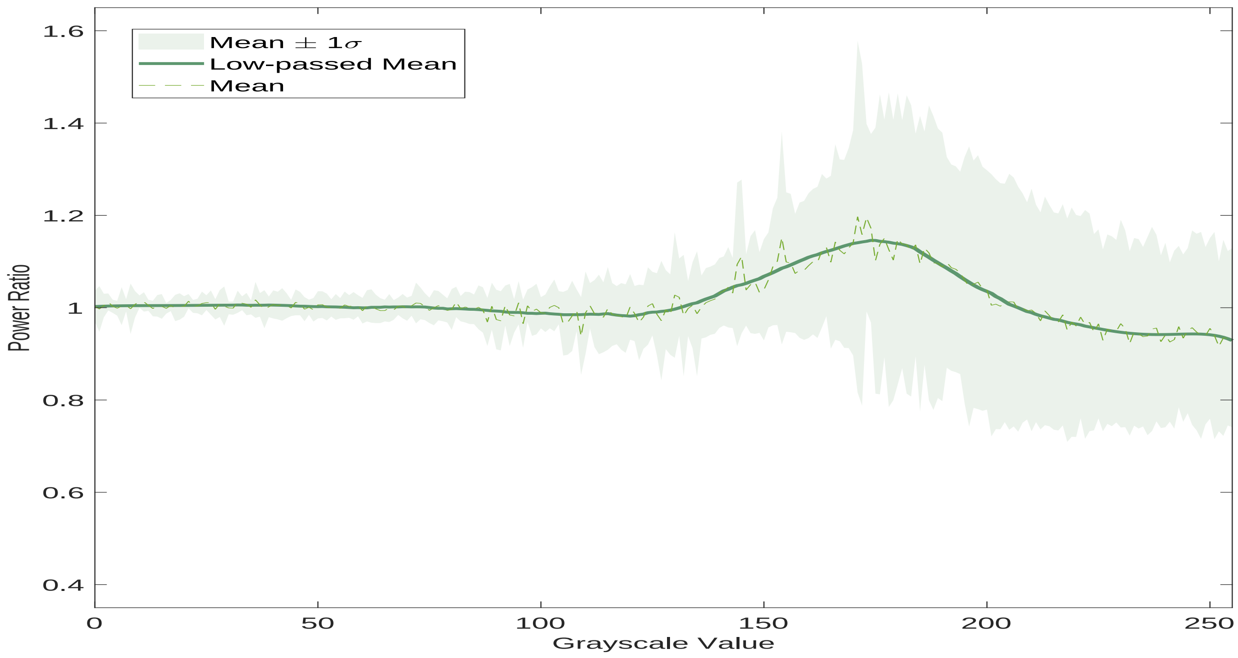

Figure S14: Phase modulation (top) and intensity modulation (below) of the new configuration of the transmissive SLM allowing larger phase depth.
